# Structure of the Inhibited State of the Sec Translocon

**DOI:** 10.1016/j.molcel.2020.06.013

**Published:** 2020-08-06

**Authors:** Samuel F. Gérard, Belinda S. Hall, Afroditi M. Zaki, Katherine A. Corfield, Peter U. Mayerhofer, Catia Costa, Daniel K. Whelligan, Philip C. Biggin, Rachel E. Simmonds, Matthew K. Higgins

**Affiliations:** 1 Department of Biochemistry, University of Oxford, Oxford OX1 3QU, UK; 2 School of Biosciences and Medicine, Faculty of Health and Medical Sciences, University of Surrey, Guildford, Surrey GU2 7XH, UK; 3 Surrey Ion Beam Centre, University of Surrey, Surrey, UK; 4 Department of Chemistry, Faculty of Engineering and Physical Sciences, University of Surrey, Guildford, Surrey GU2 7XH, UK

**Keywords:** Sec translocon, mycolactone-inhibited conformationprotein translocation, Buruli Ulcer

## Abstract

Protein secretion in eukaryotes and prokaryotes involves a universally conserved protein translocation channel formed by the Sec61 complex. Unrelated small-molecule natural products and synthetic compounds inhibit Sec61 with differential effects for different substrates or for Sec61 from different organisms, making this a promising target for therapeutic intervention. To understand the mode of inhibition and provide insight into the molecular mechanism of this dynamic translocon, we determined the structure of mammalian Sec61 inhibited by the *Mycobacterium ulcerans* exotoxin mycolactone via electron cryo-microscopy. Unexpectedly, the conformation of inhibited Sec61 is optimal for substrate engagement, with mycolactone wedging open the cytosolic side of the lateral gate. The inability of mycolactone-inhibited Sec61 to effectively transport substrate proteins implies that signal peptides and transmembrane domains pass through the site occupied by mycolactone. This provides a foundation for understanding the molecular mechanism of Sec61 inhibitors and reveals novel features of translocon function and dynamics.

## Highlights

• The inhibited Sec translocon adopts a conformation optimal for substrate engagement
• The inhibitor mycolactone wedges open the lateral gate of Sec61α
• Mycolactone blocks the path taken by the signal peptide during engagement
• Resistance mutations are likely to operate by modulating translocon dynamics

## Graphical Abstract

**Figure:**
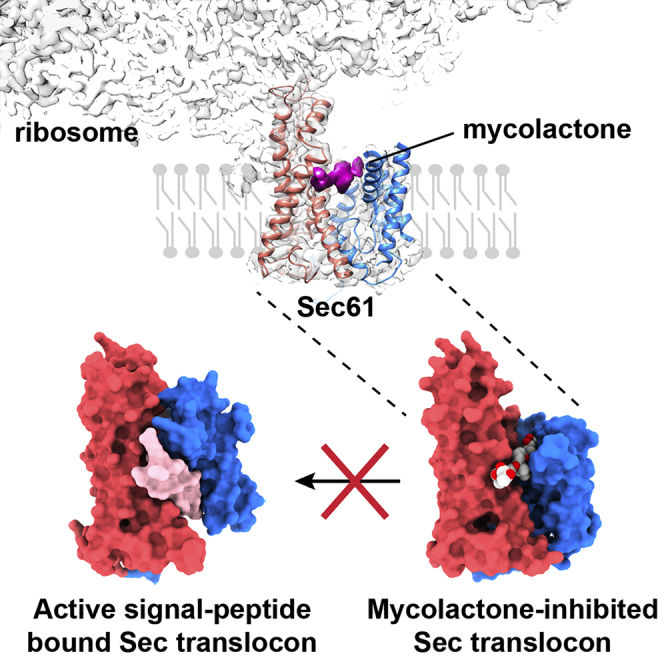

## Teaser

Gérard et al. determine the structure of the mammalian Sec translocon in an inhibited state. Mycolactone holds the translocon in a conformation optimal for substrate engagement, wedging open the cytosolic side of the lateral gate, while also blocking the path taken by the signal peptide during engagement.

## Introduction

Biogenesis of eukaryotic secretory or transmembrane proteins often occurs at the surface of the endoplasmic reticulum through the process of co-translational translocation. Here proteins targeted to the membrane by a signal sequence are translocated during translation through a membrane-spanning protein conduit formed by the Sec61αβγ complex. An analogous system, SecYEG, is found in the plasma membrane of bacteria, where it also mediates protein secretion. The structure of the heterotrimeric Sec complex is conserved throughout evolution and contains the core channel-forming subunit Sec61α/SecY together with the smaller subunits Sec61β/SecG and Sec61γ/SecE (Van den Berg et al., 2004; Voorhees et al., 2014). The translocon forms a gated channel that maintains membrane integrity while selectively opening to allow passage of the unfolded polypeptide chain. In addition, the translocon must open laterally to allow the transmembrane helices of membrane proteins to pass into the membrane environment as they are translated.

Structural studies of the translocon have provided snapshots of how this dynamic complex operates. Sec61α/SecY is formed from 10 transmembrane helices that are arranged in a “clamshell” architecture (Van den Berg et al., 2004). In the resting state, this forms a transmembrane channel, which is occluded by a central constriction of hydrophobic residues, known as the pore ring, and a short plug helix, which blocks the lumenal exit. A second potential opening, the lateral gate, lies at the interface between helices H2/H3 and H7/H8. This gate is stabilized by a polar cluster and is proposed to open to provide a route for translocating transmembrane helices to pass into the membrane (Van den Berg et al., 2004). During protein translocation, the plug helix is displaced to open the channel, whereas the dynamic opening and closing of the lateral gate is thought to allow regions of the elongating polypeptide to partition into the endoplasmic reticulum lumen or the membrane environment, depending on their hydrophobicity.

More recently, cryoelectron microscopy studies have allowed visualization of ribosome-translocon complexes in different stages of translocation. These show that ribosome binding primes the translocon, loosening the lateral gate and breaking the polar cluster (Voorhees et al., 2014). During co-translational translocation, the N-terminal signal peptide interacts with a hydrophobic patch of residues near the lateral gate, occupying the position previously taken by helix H2 of Sec61α (Voorhees and Hegde, 2016a). In this state, the plug helix is displaced, opening the channel for translocation. The translocon can also operate post-translationally, with the allosteric activators Sec62 and Sec63 stabilizing an activated conformation of Sec61α in which the cytoplasmic side of the lateral gate is open (Itskanov and Park, 2019; Wu et al., 2019) but the plug helix remains in place (Wu et al., 2019). This prepares the translocon to bind and be gated by weak signal peptides.

These studies reveal that the translocon is dynamic, with structural transitions modulated by associations with the signal peptide (Gouridis et al., 2009) or allosteric activators (Itskanov and Park, 2019; Wu et al., 2019). Open states are stabilized by binding partners that hold helices H2 and H3 in a conformation in which the lateral gate is open and the plug helix is displaced (Gemmer and Förster, 2020; Lang et al., 2017; Rapoport et al., 2017; Voorhees and Hegde, 2016b). There are also a number of inhibitors that act on Sec61α but no detailed molecular insight into how they modulate the structure or conformational dynamics of the translocon.

**Figure 1:**
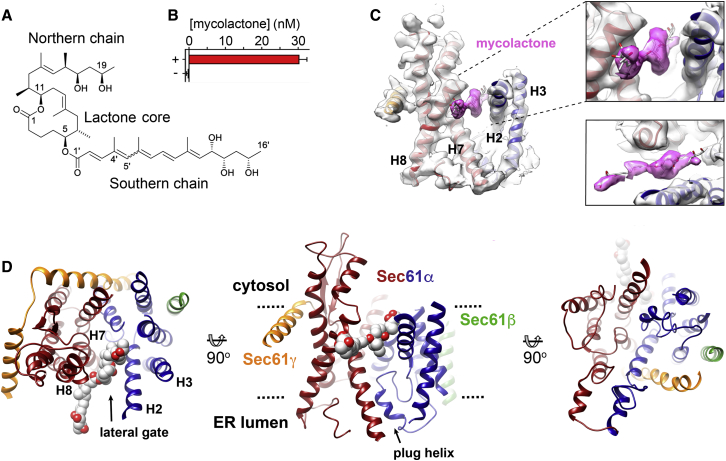
The Structure of the Sec61 Translocon Inhibited by Mycolactone (A) The structure of mycolactone A/B (743 Da). The 12-membered lactone core is indicated, as are the two polyketide side chains, commonly referred to as the northern and southern chains. Mycolactone A/B is a 3:2 rapidly equilibrating mixture of *Z*-Δ4′,5′ and *E*-Δ4′,5′ geometric isomers at the second double bond in the southern fatty acid tail. (B) Mycolactone concentrations in purified ribosome-translocon complexes extracted from membranes treated (+) or not treated (−) with mycolactone, determined by high-resolution LC-MS with the extracted ion chromatogram peaks integrated to obtain the [mycolactone+Na]^+^ (m/z 765.4721–765.5103) ion eluting at 2.38–2.98 relative to the calibration curve. (C) Electron density for Sec61α in the presence of mycolactone. Electron density maps were low-pass-filtered to 5 Å; the density feature corresponding to mycolactone is colored pink. The insets show two close-up views centered on the mycolactone density. (D) The structure of the Sec translocon with Sec61α colored, with helices H1–H5 in blue, H6–H10 in red, Sec61β in green, and Sec61γ in orange. Mycolactone is shown as spheres, with carbon in white and oxygen in red. See also Figures S1–S5.

Two studies showed that selective inhibitors of protein secretion can block co-translational translocation by acting directly on Sec61 (Besemer et al., 2005; Garrison et al., 2005). Inhibitors of Sec61-dependent translocation that act in this way include mycolactone, apratoxin A, cotransin, and ipomoeassin F (Cross et al., 2009; Hall et al., 2014; Haßdenteufel et al., 2018; Junne et al., 2015; Mackinnon et al., 2014; Paatero et al., 2016; Zong et al., 2019). These molecules have diverse structures but compete when binding to Sec61α, suggesting that they bind to overlapping sites (Baron et al., 2016; Paatero et al., 2016; Zong et al., 2019). Indeed, mutations of Sec61α that cause resistance to one inhibitor often also cause resistance to the others, suggesting a shared mode of action. Despite this, different inhibitors show variable specificity for translocons from different species and block translocation of different classes of substrates, raising the possibility of developing therapeutically useful selective translocon modulators. Of the current inhibitors, mycolactone (Figure 1A) is the most potent (Hall et al., 2014). This diffusible lipid-like exotoxin is synthesized by the Buruli ulcer pathogen *Mycobacterium ulcerans* (Demangel and High, 2018; Yotsu et al., 2018) and forms a stable complex with Sec61α (Baron et al., 2016). It prevents co-translational translocation of secretory proteins, including inflammatory mediators and cytokines, at nanomolar concentrations (Baron et al., 2016; Hall et al., 2014; McKenna et al., 2016) and blocks Sec61-dependent insertion of many transmembrane proteins (Baron et al., 2016; McKenna et al., 2017). Mycolactone inhibits translocation at a stage after ribosome engagement with the translocon and affects the interaction of signal peptides (McKenna et al., 2016). The availability of this potent and stably bound inhibitor provided us with the opportunity to visualize the translocon trapped in the inhibited state.

## Results

### Cryoelectron Microscopy of Mycolactone-Inhibited Ribosome-Translocon Complexes

Although there is, to date, no high-resolution crystal structure of a eukaryotic Sec61 homolog, cryoelectron microscopy of ribosome-translocon complexes has allowed visualization of translocons with different binding partners or captured in different states. We therefore purified ribosome-translocon complexes (RTCs) from canine microsomal membranes that had been incubated with mycolactone at a concentration that completely prevented prepro-α factor translocation (Figure S1). Liquid chromatography-mass spectrometry (LC-MS) was used to confirm the presence of mycolactone in these complexes (Figure 1B; Figure S2). In addition, to ensure that the observed changes were due to the presence of mycolactone rather than due to details of our preparation or imaging protocols, we prepared a control sample of ribosome-translocon complexes in the absence of inhibitor.

We next prepared grids to allow visualization of these samples by cryoelectron microscopy. Although samples prepared in standard holey grids or grids overlaid with a 2-nm layer of carbon showed preferred orientations, complexes vitrified on graphene oxide-coated grids allowed imaging of multiple views and generation of three-dimensional reconstructions (Figures S3–S5; Table 1). Ribosomes were predominantly translocon bound, and particle subtraction and focused classification on mycolactone-bound translocons yielded a map with an overall resolution of 2.6 Å and local resolutions for Sec61α from 2.4–8.7 Å (Figure 1; Figure S3). The resolution is highest for the C-terminal ribosome-bound half of Sec61α (H6–H10), whereas the N-terminal half (H1–H5) is more dynamic and less clearly defined (Figure S3). A model was built containing all transmembrane helices of Sec61α, the majority of Sec61γ, and a poly-alanine helix for Sec61β. The control dataset generated an electron density map at an overall resolution of 2.8 Å in which the positions of the helices were essentially indistinguishable from the previously reported structure of a primed translocon (Voorhees et al., 2014), indicating that the changes observed in the mycolactone-bound translocon were due to the presence of mycolactone (Figures S5). An elongated density within the cytosolic side of the lateral gate of the translocon was the only difference between mycolactone-bound and mycolactone-free translocons that was not due to helix movement and was attributed to mycolactone (Figure 1C). A comparison of mycolactone-bound and mycolactone-free ribosome-translocon complexes revealed no differences in the conformation of the ribosome, the occupancy of tRNA-binding sites, or the presence of associated proteins because of the presence of mycolactone (Figures S1 and S3–S5), supporting the finding that mycolactone does not directly affect translation elongation (Hall et al., 2014).

**Table 1:** Refinement and Model Statistics

-	Ribosome:Sec61 + Mycolactone	Ribosome:Sec61
**Data Collection and Processing**		

Magnification	165,000	165,000
Voltage (kV)	300	300
Electron exposure (e−/Å^2^)	49	49
Defocus range (μm)	−1.0 to −2.5	−1.0 to −2.5
Pixel size (Å)	0.822	0.822
Symmetry imposed	C1	C1
Initial particle images (no.)	108,129	49,733
Final particle images (no.)	45,733	24,852
Map resolution (Å)	2.63	2.85
Fourier Shell Correlation threshold	0.143	0.143

**Refinement**		

Map pixel size (Å)	1.033	
Map sharpening *B* factor (Å^2^)	5.7	
Map lowpass filter (Å)	4.0	
Model resolution (Å)	4.0	

**Model Composition**		

Non-hydrogen atoms	3,608	
Protein residues	441	
Ligands	MYC: 1	

**Average B Factors (Å2)**		

Protein	65.98	
Ligands	71.85	

**Root-Mean-Square Deviations (RMSDs)**		

Bond lengths (Å)	0.006	
Bond angles (°)	1.047	

**Validation**		

MolProbity score	1.79	
Clashscore	5.40	
Poor rotamers (%)	0.53	

**Ramachandran Plot**		

Favored (%)	91.61	
Allowed (%)	8.39	
Disallowed (%)	0.00	

### Mycolactone Stabilizes a Partially Activated Conformation of Sec61

**Figure 2:**
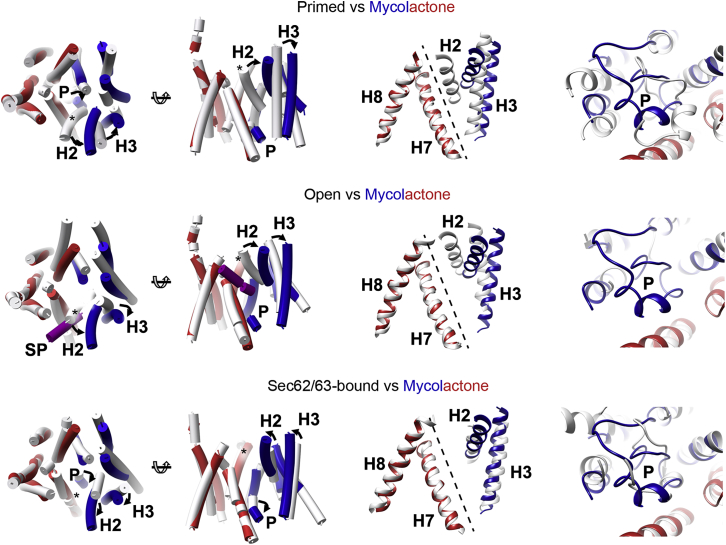
The Mycolactone-Stabilized Conformation of the Sec Translocon Structural overlays of the mycolactone-bound conformation of Sec61α, with helices H1–H5 in blue and helices H6–H10 in red. This has been overlaid with the primed conformation (top panel; PDB: 3J7Q), the open signal peptide-bound conformation (center; PDB: 3JC2), and the Sec62/63-bound conformation (bottom; PDB: 6ND1), all in white. The left panel shows the helices as cylinders. The center panel shows helices H2, H3, H7, and H8 in cartoon representation, with the lateral gate shown as a dotted line. The right panel shows a close up centered on the plug helix (P).

A comparison of the conformations of the mycolactone-bound and mycolactone-free translocons (Voorhees et al., 2014) identified no differences in the ribosome-bound C-terminal half of Sec61α. However, in the presence of mycolactone, a major structural change was observed within the more flexible N-terminal half, with an ∼9-Å movement of the cytoplasmic ends of helices H2 and H3 and smaller tilting of helix H4. This movement of H2 and H3 away from H7 and H8 results in opening of the cytoplasmic side of the lateral gate (Figure 2). Toward the lumenal side of the translocon lies the plug helix. This plug is present in the primed conformation of the translocon (Voorhees et al., 2014) but is displaced in the signal peptide-bound conformation, opening the channel (Voorhees and Hegde, 2016a). In the presence of mycolactone, the plug helix is clearly visible in the electron density maps, retaining its α-helical character but showing a displacement of ∼7 Å from its location in the primed state. However, this displacement is not sufficient to allow channel opening, with the plug helix still occluding the channel (Figure 2).

Comparison of the structure of the mycolactone-bound translocon with those of eukaryotic primed (Voorhees et al., 2014), signal peptide-bound (Voorhees and Hegde, 2016a), and Sec62/63-bound states (Itskanov and Park, 2019; Wu et al., 2019; Figure 2) reveals mycolactone to stabilize a conformation of Sec61α most similar to that observed in the presence of a complex of Sec62 and Sec63. This conformation of Sec61α is associated with post-translational translocation, in which the translocon is poised to accept substrates and to be gated by inefficient signal peptides. Indeed, stabilization of this state by Sec62/63 is thought to facilitate translocation, making it surprising that this is the state stabilized by a translocation inhibitor.

### A Proposed Binding Site for Mycolactone at the Cytoplasmic Entrance of the Channel

**Figure 3:**
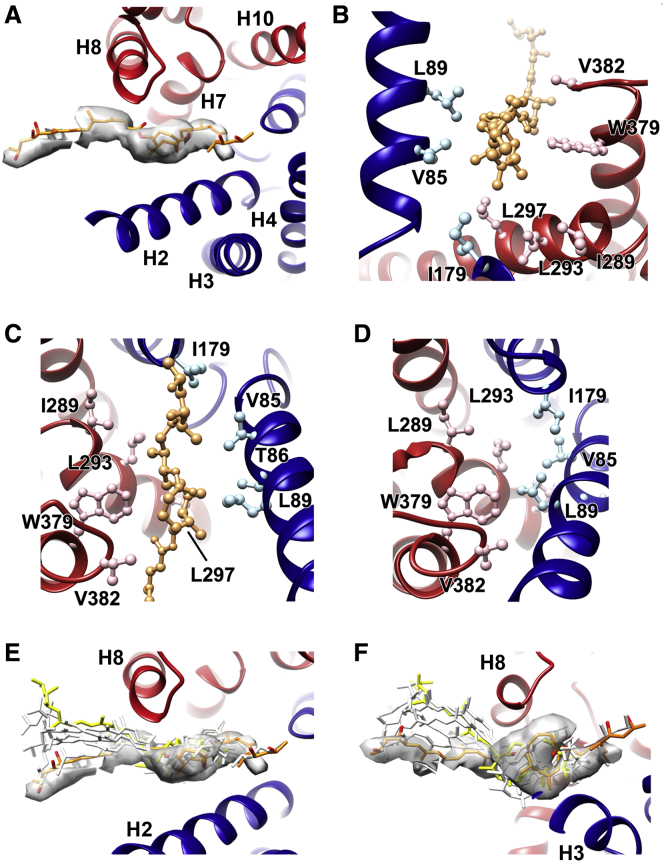
The Mycolactone Binding Site and the Location of Residues Whose Mutation Leads to Mycolactone Resistance (A) Structure of Sec61α, with helices H1–H5 in blue and H6–H10 in red. The electron density for mycolactone is shown as a white surface. (B) A close up of the mycolactone binding site, highlighting hydrophobic residues that lie in this pocket. (C) A second view of the mycolactone binding site from the cytoplasmic face, viewed approximately perpendicular to the membrane. (D) A view of the primed conformation of Sec61α, viewed from the same direction as in (C). (E and F) Two views of the outcome of molecular dynamics simulations in which mycolactone was allowed to move within a restrained translocon. The original position of mycolactone, determined by fitting into the electron density, is shown as orange sticks, whereas gray sticks show variation in the position of mycolactone through a 100-ns trajectory, and yellow sticks show the position of mycolactone at the end of the trajectory. The electron density for mycolactone is shown as a white surface. See also Figure S6.

Comparison of the electron density maps for mycolactone-bound and mycolactone-free translocons (Voorhees et al., 2014) reveals a single density feature that was not attributed to helix movement (Figure 1C). Because mycolactone must be present in the translocon to account for stabilization of this conformation, we docked mycolactone into this density. This binding pocket lies between helices H2 and H8 of Sec61α, within a hydrophobic groove created by opening of the lumenal side of the lateral gate (Figure 3A). Residues that line this groove and contact mycolactone include hydrophobic side chains that form part of the pore ring (V85 and I179) and hydrophobic patch (L89 and I179) of Sec61α and are thought to play important roles in channel closure and signal peptide binding (Figures 3B and 3C). The electron density attributed to mycolactone consists of a wider central region into which we docked the lactone core of mycolactone. On either side of this are two narrower regions of density likely to correspond to the polyketide side chains. These are not sufficiently well defined to precisely place mycolactone because of the ∼5-Å resolution of this region of the map (Figure S3) coupled with the potential for mycolactone isomerization (George et al., 1999) and the inherent flexibility of its polyketide chains.

To further assess the pose of mycolactone within this binding site and to determine whether it remains stably bound during simulation, we used molecular dynamics (Figures 3E and 3F; Figure S6). Two possible orientations of mycolactone could fit within the electron density, with the longer southern chain protruding into the hydrophobic environment of the lipid bilayer or into the core of the translocon. To assess the likelihood of each possible orientation, we modeled the *E*-Δ4′,5′ isomer of mycolactone, the presumptive active form (Gehringer et al., 2019), into this density in either possible orientation, and molecular dynamics simulations were conducted to assess their stability. In both cases, mycolactone remained stably bound over the 100-ns time course of the simulation. However, the conformation in which the southern chain protrudes into the lipid bilayer showed less variation during the simulation and was better accommodated within the binding pocket as well as within the electron density, suggesting this to be the most likely binding mode. Here the lactone core forms the majority of the interaction with the translocon, with the northern chain also interacting, whereas the long southern chain is predominantly flexible and disordered.

### Mycolactone Resistance Mutations Reduce Mycolactone Binding, Most Likely by Modulating Translocon Dynamics

**Figure 4:**
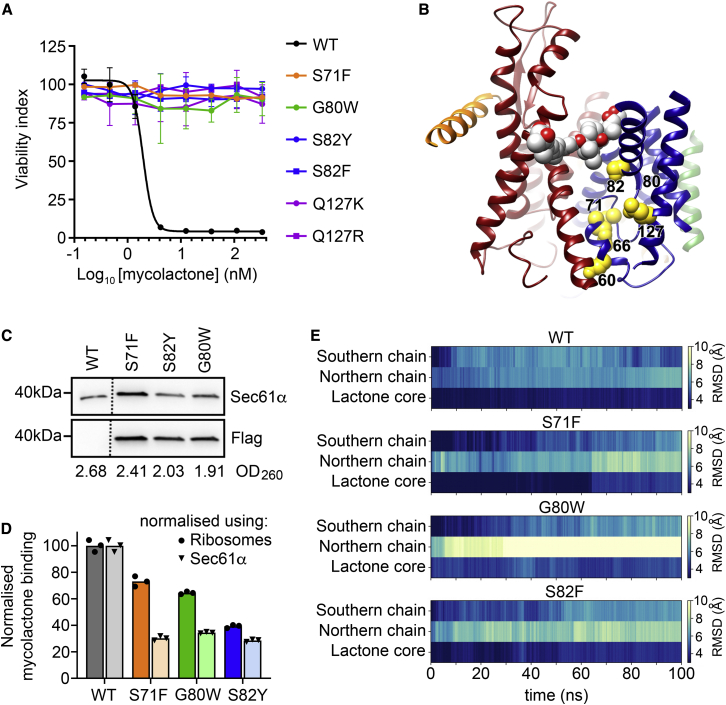
The Location of Mycolactone Resistance Mutations and Their Effect on Mycolactone Binding (A) Parental HCT-116 cells and representative clones with different amino acid substitutions were tested for their sensitivity to mycolactone A/B. Data are expressed as a normalized viability index of cells treated with inhibitor for 5 days, after which metabolic activity was assessed with Resazurin dye (alamarBlue assay), and values were normalized to a DMSO control. The IC_50_ of wild-type cells for mycolactone A/B was 1.94 nM (1.44 ng/mL). Data are mean ± SEM of n = 3. (B) A representation of the Sec translocon with Sec61α colored, with helices H1–H5 in blue and H6–H10 in red, Sec61β in green, and Sec61γ in orange. Mycolactone is shown as spheres, with carbon in white and oxygen in red. Residues whose mutation leads to mycolactone resistance are represented as yellow spheres. (C and D) Microsomes were prepared from TRex-293 cells or those stably transfected with C-terminal FLAG-tagged mutant Sec61A1 constructs. Mycolactone-exposed RTCs were prepared, and the peak fractions were subjected to LC-MS. (C) Immunoblotting of peak fractions. Migration relative to known molecular weight markers is shown, as is optical density 260 (OD_260_). The location of an excised lane is indicated by a dotted line. (D) Relative mycolactone abundance in the RTCs was estimated using high-resolution LC-MS by integrating the extracted ion chromatogram peaks for the [mycolactone+Na]^+^ (m/z 765.4721–765.5103) ion eluting at 2.38–2.98 relative to a calibration curve. To compare between preparations, the data were standardized on ribosome concentration (calculated from OD_260_) or Sec61α abundance (pixel intensity of the immunoblot band). Triplicate analysis from n = 1. (E) Assessment of the dynamics of mycolactone bound to the translocon during 100-ns simulations for wild-type and mutant translocons. Helices H6–H10 were restrained, and movement was allowed for mycolactone and the remainder of the translocon. In each case, the root-mean-square deviation from the starting position is shown at each nanosecond for the southern chain, northern chain, and lactone core. Three independent simulations were conducted, and these plots show the average. See also Figures S7–S9 and Tables S1 and S2.

The structure of the mycolactone-bound translocon allowed us to revisit how Sec61α mutations cause resistance to inhibitors. A number of studies have identified mutations that overcome the cytotoxic effects of mycolactone. Indeed, the same mutations often affect multiple different inhibitors, supporting the idea of a common binding mode (Baron et al., 2016; Junne et al., 2015; Mackinnon et al., 2014; Ogbechi et al., 2018; Paatero et al., 2016; Zong et al., 2019). We now conducted an additional forward genetic screen that identified nine substitutions in six codons that gave mycolactone resistance, five of which overlapped with those identified previously (Figure 4A; Figure S7; Tables S1 and S2). Although not identified in our screen, mutation of T86 is also reported to confer resistance to mycolactone (Zong et al., 2019). Unexpectedly, of the residues associated with resistance mutations, only T86 directly contacts mycolactone. Instead, the majority lie on or around the plug helix, on the lumenal side of the translocon, away from the mycolactone-binding pocket (Figure 4B). Indeed, no extra electron density is observed in the mycolactone-bound translocon, which directly contacts residues associated with resistance mutations.

Although we cannot be sure why resistance mutations were not found in the binding pocket, this may be due to the essential role of residues that contact mycolactone. These include hydrophobic residues that play functionally important roles in the structure and dynamics of Sec61α, including roles in the pore ring (V85 and I179) and the hydrophobic patch (L89 and I179). It is therefore possible that mutations in these residues that cause sufficiently large changes in side-chain chemistry to reduce mycolactone binding also make the translocon non-functional, precluding their appearance in a forward genetic screen. Indeed, the naturally occurring mutation V85D, found in hypogammaglobulinemia, is defective in co-translational translocation (Schubert et al., 2018).

With the surprising finding that resistance mutations are not found in residues that contact mycolactone, we used LC-MS and molecular dynamics to assess whether these mutations affect binding of mycolactone to the translocon. To directly measure mycolactone binding, we prepared microsomes from unaltered TRex-293 cells as well as from cells stably overexpressing Sec61α with resistance mutations at positions S71, G80, and S82. RTCs were prepared in the presence of mycolactone, and LC-MS was used to quantify mycolactone in these complexes. In wild-type cells, we estimate that each translocon bound an average of 0.87 ± 0.04 mycolactone molecules. When performed with cells overexpressing mutant Sec61α variants, in each case, the average number of mycolactone molecules per translocon decreased, with reductions of 70%–80% when normalization was conducted using a Sec61α immunoblot (Figures 4C and 4D; Figure S8). Because endogenous Sec61α remains in these preparations, this shows that all three mutations cause major reductions in the binding of mycolactone to the translocon.

In parallel, we performed molecular dynamics to assess the stability of mycolactone in the binding pocket. Long simulations of the entire ribosome-translocon system to observe Sec and mycolactone conformational dynamics were computationally prohibitive. However, short MD simulations can successfully be used to provide an assessment of binding stability (Liu et al., 2017). Because helices H6–H10 of the translocon were equivalent in conformation between mycolactone-bound and -free conformations (Figure 2), we fixed these in position and allowed free movement of mycolactone and helices H1–H5. In three repeats of a 100-ns simulation, mycolactone remained stably bound. However, introduction of the S71F, G80W, and S82F mutations each reduced the stability of mycolactone binding, in particular of the northern chain (Figure 4E; Figure S9). Together with MS analysis, these studies show that resistance mutations decrease mycolactone binding despite being in residues that do not make direct contact with mycolactone.

Therefore, the most probable hypothesis for how resistance mutations function is that they modulate translocon dynamics, reducing the likelihood of formation of the mycolactone binding pocket. To assess this, we measured the change in separation of three pairs of residues that contact mycolactone, V85-W379, L89-V382, and L89-W379, during the molecular dynamics simulations of mycolactone-bound S71F, G80W, and S82F described above (Figure S9). Intriguingly, each mutation changed the separation of these residues in different ways. S82F seems to favor an opening of the lateral gate in the region of the mycolactone binding pocket, perhaps because of introduction of a bulky side chain in a site buried in the mycolactone-bound conformation. In contrast, G80W favored closing of the lateral gate. A conclusive analysis would require extensive simulations of complete RTCs and detailed biochemical analyses of the mutant translocons. However, our analysis suggests that favoring the open or closed state relative to the mycolactone-bound state can be associated with resistance. Indeed, a similar allosteric mechanism has been proposed to explain the role of the *prl* phenotypes that allow export of proteins with defective or absent signal peptides (Junne et al., 2007, 2015; Smith et al., 2005; Trueman et al., 2012). Most of the residues in which we identified resistance mutations are also associated with *prl* phenotypes (Table S1), supporting the idea that their effects are the result of changes in translocon dynamics. Our findings therefore confirm that resistance mutations reduce mycolactone binding without being in residues that directly contact the inhibitor and suggest that this is achieved by changes in conformational dynamics that disfavor the mycolactone-bound conformation.

## Discussion

This study reveals the mode of action of Sec61α inhibitors while also highlighting unexpected features of the mechanism of the translocon. First we show that mycolactone wedges open the cytoplasmic side of the lateral gate of Sec61α. This stabilizes a conformation very similar to that stabilized by Sec62/63, which is poised and permissive for post-translational translocation. Therefore, unexpectedly, mycolactone does not stabilize the inactive translocon but traps the helices of Sec61α in a partly activated conformation.

The density attributed to mycolactone lies directly across the cytoplasmic side of the channel. Although the route the signal peptide takes during co-translational translocation is not fully resolved, the location of the engaged signal peptide has been mapped to a site within the lateral gate in translocating ribosome-translocon complexes (Voorhees and Hegde, 2016a). The locations occupied by mycolactone and the signal peptide overlap and will be mutually exclusive in occupation. This suggests that the signal peptide reaches its binding site by passing through the region occupied by mycolactone and that the inhibitor directly prevents signal peptide-mediated opening of the channel and subsequent removal of the plug.

**Figure 5:**
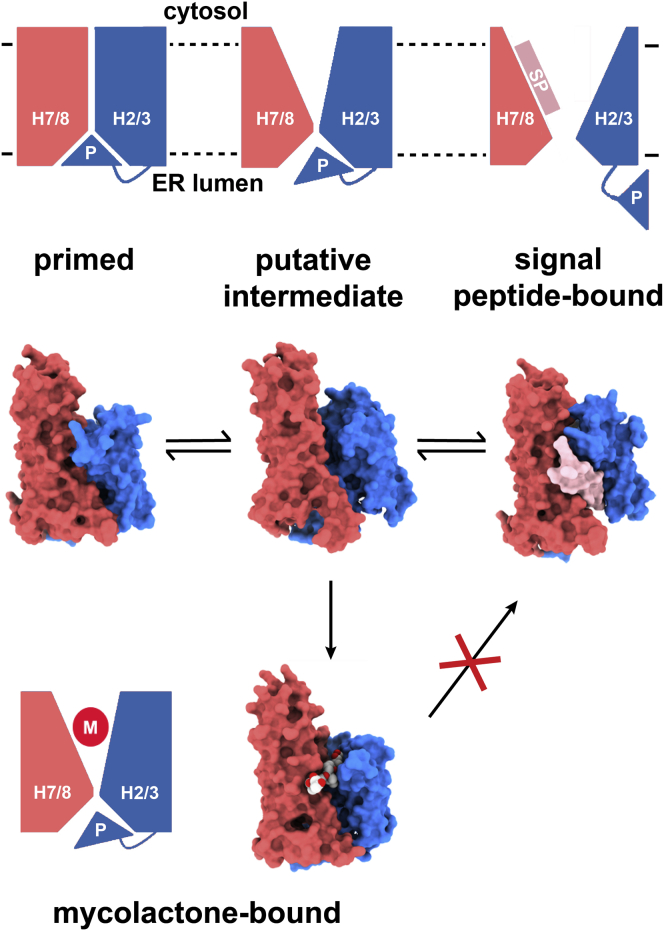
A Model of the Mode of Action of Mycolactone In this model, the ribosome-bound primed conformation of the Sec translocon is hypothesized to be in equilibrium with a second state (the putative intermediate), in which the lateral gate is open while the plug helix blocks the channel. This is similar in structure to the state stabilized by Sec62/63. Mycolactone can enter this intermediate state and stabilize its conformation while hindering access to the signal peptide binding site.

These findings also have consequences for our understanding of the dynamics of the translocon (Figure 5). The binding site for mycolactone will only be formed when the cytoplasmic side of the lateral gate opens through the tilting of helices H2 and H3 away from H7 and H8. It is hard to envisage mycolactone occupying this binding pocket when Sec61α is in the process of translocation because the translocating translocon will be open, and the nascent polypeptide chain is expected to occlude the mycolactone binding site. Indeed, this is supported by biochemical assays showing that mycolactone does not affect cross-linking of a substrate to Sec61α when added after translocation is initiated by microsome addition (McKenna et al., 2016). Instead, mycolactone will bind while the translocon is idle. This suggests that non-translocating Sec61α is in a dynamic equilibrium, transitioning between the closed state and a transient intermediate that resembles the inhibitor-bound state. This conformational mobility will be advantageous for the translocon because the plug helix remains in place, keeping the channel of the translocon closed to minimize ion leak, but movements in the lateral gate create an opportunity for signal peptide binding and gating. The finding that Sec62/63 stabilizes this intermediate to facilitate post-translational translocation supports this view. However, this intermediate is also susceptible to inhibition, with transient opening of the lateral gate creating the inhibitor binding pocket, allowing mycolactone to wedge open the lateral gate and block access to the signal peptide binding site.

Translocation inhibitors with profoundly different chemical structures show cross-competition and are affected by the same resistance mutations (Van Puyenbroeck and Vermeire, 2018), suggesting that this mechanism is not restricted to mycolactone but common and widely exploitable. Indeed, our findings may also explain why the same resistance mutations affect the efficacy of inhibitors as diverse in structure as cyclic peptides, glycolipids, and polyketide lactones because they modulate formation of their shared binding site. Translocation inhibitors have been suggested as potential anti-cancer and anti-inflammatory agents (Van Puyenbroeck and Vermeire, 2018) and would be expected to have powerful activity against enveloped viruses. This structure not only yields new insight into the mechanism of the translocon but also provides invaluable insight to guide future rational drug design.

## STAR★Methods

### Key Resource Table

**Table:** 

REAGENT or RESOURCE	SOURCE	IDENTIFIER
**Antibodies**		

Mouse monoclonal anti-Sec61α	Santa Cruz Biotechnology	Cat#sc-393182, RRID: AB_2301616
Rabbit polyclonal anti-Sec61β	Kelkar and Dobberstein, 2009	N/A
Rabbit polyclonal anti-RS6	Cell Signaling Technology	Cat#2217, RRID: AB_331355
Mouse monoclonal anti-Ribophorin 2	Santa Cruz Biotechnology	Cat#sc-166421 RRID: AB_2238716
DYKDDDDK Tag Rabbit anti-Tag, Polyclonal	Invitrogen	Cat# PA1984B RRID: AB_347227
ECL Mouse IgG, HRP-linked	GE Healthcare	Cat#NXA931 RRID: AB_772209
ECL Rabbit IgG, HRP-linked	GE Healthcare	Cat#NA934 RRID: AB_772206
Goat Anti-Rabbit IgG (H+L) Antibody, Alexa Fluor 488 Conjugated	Invitrogen	Cat# A-11008 RRID: AB_143165

**Chemicals, Peptides, and Recombinant Proteins**		

Mycolactone A/B	Song et al., 2002	CAS:222050-77-3
L-[S35]-Methionine	Hartmann Analytic	Cat#SCM01
Digitonin	Sigma-Aldrich	Cat#D141, CAS:11024-24-1
Cotransin	MacKinnon et al. 2007; Mackinnon et al. 2014	CAS: 1000770-96-6
Ipomoeassin F	Cao et al. 2007; Zong et al., 2019	CAS:915392-44-8
Resazurin sodium salt	Sigma Aldrich	Cat#R7017, CAS:62758-13-8
Mycolactone A/B, mixture of epimers at C12	Saint-Auret et al., 2017a, 2017b	N/A
Paraformaldehyde	Sigma Aldrich	Cat#P6148

**Deposited Data**		

Canine ribosome:Sec61 complex cryo-EM map in the presence of mycolactone	This paper	EMDB: 11064
Canine Sec61 channel bound to mycolactone model coordinates	This paper	PDB: 6Z3T
Canine ribosome:Sec61 complex cryo-EM map in the absence of mycolactone	This paper	EMDB: 11064
Original images and immunoblots, Sanger sequencing, viability assays and LC-MS data	This paper	https://data.mendeley.com/datasets/cc92fyz9sv/1

**Experimental Models: Cell Lines**		

HCT-116 cell line	ATCC	CCL-247
TRex-293 cell line	Thermo Fisher	R71007
TRex-293 Sec61α S71F C-terminal Flag	This paper	N/A
TRex-293 Sec61α G80W C-terminal Flag	This paper	N/A
TRex-293 Sec61α S82Y C-terminal Flag	This paper	N/A

**Oligonucleotides**		

Sec61A1_frag1F TAGCACTGACGTGTCTCTCG	Sigma Aldrich	N/A
Sec61A1_frag1R TCCCCATACATCCCGGTCAT	Sigma Aldrich	N/A
Sec61A1_frag2F CTTCAACGGAGCCCAAAAGT	Sigma Aldrich	N/A
Sec61A1_frag2R GTGTTGTACTGGCCACGGTAG	Sigma Aldrich	N/A
Sec61A1_frag3F TCATCGCCACCATCTTTGTCTT	Sigma Aldrich	N/A
Sec61A1_frag3R GGACCATGGAGGTCTCTCGG	Sigma Aldrich	N/A
Sec61A1_frag4F TATACATAGTGTTCATGCTGGGCT	Sigma Aldrich	N/A
Sec61A1_frag4R ACACAGTGGAATGAAAGAATACGA	Sigma Aldrich	N/A
Sec61A1_frag5F TAGTGTGCTGCCAGATTCCC	Sigma Aldrich	N/A
Sec61A1_frag5R TCAAATTCCATTCCTCGGCCA	Sigma Aldrich	N/A

**Recombinant DNA**		

pMXSec61S71F	ThermoFisher Scientific GeneArt	N/A
pMXSec61G80W	ThermoFisher Scientific GeneArt	N/A
pMXSec61S82Y	ThermoFisher Scientific GeneArt	N/A
pNLF1-C (CMV/Hygro)	Promega	Cat#N1361
pNSec61S71F	This paper	N/A
pNSec61G80W	This paper	N/A
pNSec61S82Y	This paper	N/A

**Software and Algorithms**		

Coot	Emsley and Cowtan, 2004	https://www2.mrc-lmb.cam.ac.uk/personal/pemsley/coot/
Phenix	Adams et al., 2010	https://www.phenix-online.org/
UCSF Chimera	Pettersen et al., 2004	https://www.cgl.ucsf.edu/chimera/
UCSF ChimeraX	Goddard et al., 2018	https://www.cgl.ucsf.edu/chimerax/
Relion 3.0	Zivanov et al., 2018	https://www3.mrc-lmb.cam.ac.uk/relion/index.php/Main_Page
UCSF MotionCor2	Zheng et al., 2017	https://emcore.ucsf.edu/ucsf-motioncor2
CTFFIND 4.1	Rohou and Grigorieff, 2015	https://grigoriefflab.umassmed.edu/ctffind4
SIMPLE	Elmlund and Elmlund, 2012	https://simplecryoem.com
ResMap	Swint-Kruse and Brown, 2005	http://resmap.sourceforge.net
EPU 2.3	ThermoFisher Scientific	N/A
Adobe Illustrator	Adobe	N/A
Xcalibur 4.2 Quan Browser	Thermo Scientific	N/A
Chromas Lite	Technelysium	
BLASTn	NCBI	https://blast.ncbi.nlm.nih.gov/Blast.cgi
Prism 8.2.1	GraphPad	N/A
Excel 2016	Microsoft	N/A
Powerpoint 2016	Microsoft	N/A
ImageJ 1.52p	NIH	https://imagej.nih.gov/ij/
GROMACS 2019	Van Der Spoel et al., 2005	http://www.gromacs.org/
VMD 1.9	Humphrey et al., 1996	https://www.ks.uiuc.edu/Research/vmd/
MDAnalysis	Michaud-Agrawal et al., 2011	https://www.mdanalysis.org/
Python		https://www.python.org/
Matplotlib	https://doi.org/10.5281/zenodo.2577644	https://matplotlib.org/
PyMOL	The PyMOL Molecular Graphics System, Version 1.8 Schrödinger, LLC.	https://pymol.org/2/

**Other**		

Sephacryl S-300 HR	GE Healthcare	Cat#17059910
Micrococcal nuclease	New England Biolabs	Cat#M0247S
Rabbit reticulocyte lysate, nuclease treated	Promega	Cat#L4960
Albumin,bovine serum, protease free	ThermoFisher Scientific	Cat#11461655
Graphene oxide dispersion	Graphenea	Cat#947-768-1
Quantifoil R 1.2/1.3 200 Mesh, copper	Quantifoil	Cat#Q2100CR1.3
4–20% TGX Mini-Protean precast gels	BioRad	Cat#4561903
Spectra broad-range protein markers	Thermo Fisher Scientific	Cat#266234
Simply Blue	Thermo Fisher Scientific	Cat#10432072
Immobilon-P PVDF membrane	Thermo Fisher Scientific	Cat#15750319
Immobilon Western chemiluminescent substrate	Sigma Aldrich	Cat# WBKLS
McCoy’s 5A (modified) Medium	ThermoFisher Scientific	Cat#11530646
Foetal Bovine Serum, heat inactivated, SA origin	ThermoFisher Scientific	Cat#11550356
Superscript IV	Life Technologies	Cat# 18090050
GoTaq G2 Green Mastermix	Promega	Cat# M7822
Dulbecco’s Modified Eagle’s Medium - high glucose	Sigma Aldrich	Cat#D6429
Fugene 6	Promega	Cat#E2691
Protease inhibitor cocktail	Sigma Aldrich	Cat#P8340

### Resource Availability

#### Lead Contact

Further information and requests for resources and reagents should be directed to and will be fulfilled by the Lead Contact, Rachel Simmonds rachel.simmonds@surrey.ac.uk

#### Materials Availability

All unique/stable reagents generated in this study (plasmids containing mutant human Sec61α and mycolactone-resistant cell lines) are available from the Lead Contact without restriction.

#### Data and Code Availability

The electron microscopy datasets generated during this study, in the form of maps, are available at the electron microscopy data bank (EMD-11064). The model for the Sec translocon with mycolactone bound, generated during this study, is available at the protein data bank PDB: (6Z3T). Other data associated with the manuscript is available at https://data.mendeley.com/datasets/cc92fyz9sv/1.

### Experimental Model and Subject Detail

#### Cell lines

The male human colorectal cancer cell line HCT-116 (ATCC CCL-247) was maintained in McCoy’s 5A (modified) medium supplemented with 10% fetal bovine serum (FBS) and 1x penicillin-streptomycin (both Thermo Fisher Scientific). TRex-293 cells, derived from the female human embryonic kidney line HEK293, were maintained in high glucose DMEM, supplemented with 10% FBS. Cells were routinely cultured at 37°C and 5% CO2. We do not routinely authenticate our cell lines.

### Method Details

#### Mycolactone

We used synthetic mycolactone A/B, kindly provided by Prof Yoshito Kishi (Harvard University) (Song et al., 2002) for all of the experiments described in this manuscript. Mycolactone A/B is a 3:2 rapidly equilibrating mixture of *Z*-Δ^4’,5′^ (A) and *E*-Δ^4’,5′^ (B) geometric isomers at the second double bond in the southern fatty acid tail. Since the two isomers cannot be separated in light, as they rapidly re-equilibrate to the 3:2 ratio, we refer to them as mycolactone. Mycolactone was provided in ethyl-acetate, dried down under nitrogen and resuspended in DMSO at 0.5 mg/ml. Aliquots are protected from light and stored at −80°C.

For selection of stably transfected TRex-293 cells to derive overexpressing mycolactone resistance mutations, we used synthetic mycolactone A/B as a mixture of epimers at C12′, kindly provided by Dr Nicolas Blanchard (Saint-Auret et al., 2017a, 2017b) (French National Centre for Scientific Research), which is available in the greater amounts needed for cells under selection. However, tests performed with stable clones used mycolactone A/B (Song et al., 2002).

#### Purification of ribosome-translocon complexes

Ribosome-associated Sec61 complexes, known as ribosome-translocon complexes or RTCs, were prepared largely as described (Voorhees et al., 2014). Briefly, rough microsomal membranes (CPMM) were prepared from dog pancreas as described (Walter and Blobel, 1983) and stored at −80°C in 50 mM triethanolamine (pH 7.5), 250 mM sucrose, 1 mM DTT. Aliquots were incubated with 1 mM CaCl_2_ and 150 U/ml micrococcal nuclease for 7 min at room temperature. The reaction was stopped by addition of 2 mM EGTA and microsomes were flash-frozen in 50 μl aliquots. Mycolactone (1 μg/ml) was prepared by dilution in 0.1% (w/v) BSA from a concentrated stock. Control samples contained 0.1% (v/v) DMSO in BSA. Microsomes were thawed on ice and mycolactone was added to 200 ng/ml (269.2 nM). After gentle mixing, samples were incubated on ice for 30 min. Microsomes were solubilised by incubation for 10 min on ice with an equal volume of 100 mM HEPES pH 7.5, 800 mM KOAc, 20 mM MgOAc, 3.5% (w/v) digitonin, 2 mM DTT. Samples were centrifuged at 20,000 x g for 15min at 4°C. An aliquot of supernatant (5μl) was removed for LC-MS analysis and the rest was applied to a 1 mL Sephacryl-300 column pre-equilibrated with ice cold column buffer (50mM HEPES pH7.5, 200 mM KOAc, 10mM MgOAc, 0.25% (w/v) digitonin, 1 mM DTT). Samples were eluted at 4°C in column buffer and collected in approximately 100 μl fractions. Absorbance at 260 nm was estimated using a Nanodrop (Thermo Fisher) and the initial peak eluting fractions were pooled. Samples were re-centrifuged at 20,000 x g for 15 min at 4°C and processed immediately for electron cryo-microscopy.

#### *In vitro* translation/translocation assays

*In vitro* translation/translocation assays were performed as previously described (Hall et al., 2014). Briefly, mycolactone was added to micronuclease-treated canine pancreatic microsomes and incubated on ice for 20 min in the dark. *In vitro* translation reactions were carried out in a 25 μl volume using nuclease-free rabbit reticulocyte lysates (Promega) according to manufacturer’s instructions, with 0.5 μg prepro-α Factor mRNA and 35-S methionine. Microsomal membranes, when present, made up 10% of the final volume of the reaction mix. Samples were incubated for 30 min at 30°C and the reaction was stopped with the addition of an equal volume of 2x Laemmli sample buffer. Proteins were separated by SDS-PAGE on 4%–15% acrylamide gels, fixed, stained, and dried, then visualized with a Typhoon Phosphorimager (GE Healthcare).

#### Liquid chromatography mass spectrometry

Liquid chromatography mass spectrometry (LC-MS) was performed on a Thermo Scientific Ultimate3000 UHPLC system equipped with a binary solvent manager, column manager, and autosampler, coupled to a Thermo Orbitrap Q-Exactive Plus mass spectrometer operating with the standard electrospray ionization interface and the following conditions: spray voltage 4 kV, capillary temp. 375°C, sheath gas 60 psi, aux gas 20 psi, S lens RF level 100%, resolution 70,000, divert valve schedule (to divert sample buffer components away from MS): 0-2.3 min, waste; 2.3-3.0 min, MS; 3.0-5.0 min, waste. Liquid chromatography conditions were as follows: injection volume 10 μL; column: Phenomenex Luna Omega (C18 porous silica, 2.1 × 50 mm, 5 μm particle size, 100 Å pore size); column temp. 25°C, flow rate 0.5 mL min^-1^; mobile phase Solvent A: aqueous ammonium acetate (10mM), pH 6.9; Solvent B: acetonitrile; gradient 0-1 min, 5% B; 1-2 min, 5%–90% B; 2-3.5 min, 90% B; 3.5-4.5 min,90%–5% B; 4.5-5 min, 5% B; autosampler temp. 10°C.

The identity and LC-MS retention time of pure mycolactone A/B bound to microsomes was confirmed by both accurate mass and MS/MS of the [M+Na]^+^ ion which showed the same fragmentation pattern of sodium adducts reported previously (Hong et al., 2003) (core + northern chain *m/z* 429; southern fatty acid *m/z* 359).

For mycolactone quantification, a set of standards for an LC-MS calibration curve were set up using synthetic mycolactone A/B dissolved into acetonitrile/water (1:1). The standards were analyzed by LC-MS once before each repeat of experimental samples. Extracted ion chromatograms (EIC) for mass range 765.4721 – 765.5103 (mycolactone A/B [M+Na]^+^) were produced using Thermo Scientific Xcalibur 4.2 Quan Browser. Peak areas were first generated automatically for retention time range 2.68 min with window 36 s (2.38 – 2.98 min), ICIS peak integration using smoothing points 7, baseline window 40, area noise factor 5, peak noise factor 10. This was followed by manual adjustment of the integration to include the entire EIC peak.

Experimental samples were: freshly prepared stocks of mycolactone (13.46 μM; 10 μg/ml) in 0.1% (w/v) BSA diluted 1:50 or 1:500 in acetonitrile/water (1:1), total extract from microsomes incubated with or without mycolactone diluted 1:10 in acetonitrile/water (1:1), and pooled Sephacryl-300 peak fractions containing RTCs (undiluted). Each sample was run three times with blank runs in between. Mycolactone concentrations were determined relative to the calibration curve.

#### SDS-PAGE and immunoblotting

Aliquots of column eluted fractions were separated by SDS-PAGE on 4%–20% acrylamide gradient gels (Bio-Rad) alongside Spectra broad-range protein markers (Thermo Fisher Scientific). Proteins were stained with Simply Blue (Thermo Fisher Scientific) or blotted onto PVDF membranes. After transfer, blots were fixed with 5% acetic acid and blocked with 5% milk. Primary antibodies used were: mouse monoclonal anti-Sec61α; rabbit polyclonal anti-Sec61β (Kind gift from the Dobberstein lab) (Kelkar and Dobberstein, 2009); rabbit polyclonal anti-ribosomal protein S6 and mouse monoclonal anti-ribophorin 2 (Santa Cruz Biotechnology, sc-166421). Secondary antibodies were ECL Mouse IgG, HRP-linked (GE Healthcare, NXA931) and ECL Rabbit IgG, HRP-linked (GE Healthcare, NA934). Western blots were incubated with Immobilion Chemiluminescent HRP substrate and imaged using a Vilber Fusion FX imaging system. Images presented here were inverted using ImageJ v.1.52p.

#### Grid preparation and data acquisition

Carbon-coated holey grids (Quantifoil, R1.2/1.3, 200 mesh) were used for the preparation of graphene-oxide grids. The grids were plasma-cleaned using a Quorum device at 50 mA for 60 s. For each grid, 3 μL of 0.2 mg/ml graphene oxide dispersion (Graphenea) was applied on the carbon surface, manually blotted, and allowed to dry overnight.

A 4 μL sample with an absorbance of 10 at 260nm was applied on the surface of each grid and vitrified by plunge-freezing in liquid ethane using a Vitrobot (FEI) with an incubation time of 15 s, a blotting time of 5 s and a blotting force of −15 at 4°C.

Data were collected manually using the acquisition software EPU on a Titan Krios transmission electron microscope (FEI) at a defocus range between 1 and 2.5 μm. All data were recorded on a K2 detector under low dose conditions with a nominal pixel size of 0.822 Å/pixel on the object scale. A total of 5062 and 3833 micrographs were collected for RTCs with and without mycolactone, respectively. Each micrograph experienced a total exposure of ∼49 electrons per Å^2^ fractionated into 32 frames. Foil holes containing the thinnest regions of ice and darkest edges were manually selected as they mostly contained single layer graphene oxide and showed higher signal-to-noise ratio.

#### Image processing

Original movie frames were motion corrected and combined using MotionCor2 (Zheng et al., 2017). The contrast transfer function parameters were estimated via CTFFIND4.1 (Rohou and Grigorieff, 2015). All micrographs were screened manually for graphene oxide and ice quality. Particles were picked automatically with SIMPLE (Elmlund and Elmlund, 2012). All classifications and refinements were performed using Relion3 (Scheres, 2012; Zivanov et al., 2018). After reference-free 2D classification, particles (76,650 for RTCs with mycolactone, 35,366 for the control dataset) were subjected to an extensive 3D classification into 10 classes after an initial round of refinement.

The main differences between the three-dimensional classes was the absence or presence of eEF2, while most ribosomes contained tRNA in P/E-state or E-state. Two main classes were selected, which included ribosomes with eEF2 (32,456 particles in the presence of mycolactone, 8,607 in the control dataset) and without eEF2 (18,365 particles with mycolactone, 16,245 without). Comparison of these ribosome states with those seen by Voorhees et al. (2014), who used a similar protocol for purification of ribosome-translocon complexes, revealed similarities and differences. Voorhees observed 13% active ribosomes (with A/P and P/E-site tRNAs), 65% without tRNA but with eEF2 and 22% empty (Voorhees et al., 2014). The Voorhees et al. (2014) data share with our findings the presence of a majority of idle ribosomes and the surprising presence of a large fraction containing elongation factor eEF2. The major difference in ribosome conformation when compared with Voorhees is the presence of E or P/E site tRNAs in our RTCs, while Voorhees et al. (2014) observed predominantly tRNA-free ribosomes. We are not sure of the reasons for the differences here, but they might be due to different sources of microsomes (porcine versus canine) or subtle differences in the preparation procedure or time taken for purification.

For each state, 60S-Sec61 masked refinement, followed by particle subtraction of the ribosome core, were carried out. The subtracted particles were used for focused 3D classification with a mask applied around Sec61. The best classes were selected and subjected to 60S-Sec61 masked 3D refinement after particle polishing and CTF refinement. Particles from the two reconstructions obtained were combined, as no differences were observed in the conformation of the translocon, followed by a final 60S-Sec61 masked refinement. In the case of the control dataset, the same pipeline was used, without focused classification. All final reconstructions were subjected to post processing using a wide soft edge mask. This resulted in final resolutions of 2.63Å and 2.85Å, according to the FSC 0.143 criterion following the Relion gold-standard refinement (Scheres and Chen, 2012). Local resolution at the translocon was estimated using ResMap (Swint-Kruse and Brown, 2005). For better visualization and interpretation of the Sec density, and due to the heterogenous resolution across the translocon, the map used for model building was lowpass-filtered to 4Å.

#### Homology modeling and refinement of atomic models

The structure of Sec61 was built using the structures of the primed state (RCSB code 3J7Q) and that of the signal peptide-engaged state (RCSB code 3JC2) as reference models. The model was built in COOT (Emsley and Cowtan, 2004) and refined using Phenix (Adams et al., 2010) using Ramachandran and secondary structure restraints.

#### Molecular dynamics simulations

The final model derived from electron cryo-microscopy was used as the starting point for molecular dynamics simulations. The original conformation had mycolactone docked with the southern chain projecting toward the lipid phase while in the reversed conformation, the southern chain projected toward the translocon. The presumptive active form, the *E*-Δ^4'5^' isomer, was selected for the simulations. The parameters for mycolactone were taken from (López et al., 2018), who developed them using the General Amber force field (GAFF) (Wang et al., 2004) in combination with the restrained electrostatic potential (RESP) approach (Bayley et al., 1993) employed for optimization of partial charges. The Amber 99SB-ILDN force field (Lindorff-Larsen et al., 2010) was adopted for Sec61 in conjunction with the TIP3P water model (Jorgensen et al., 1983). The POPC lipids were represented with the Slipids force field (Jämbeck and Lyubartsev, 2012a, 2012b, 2013).

The Sec61/mycolactone complex was first oriented with the translocon pore axis aligned coincident with the membrane normal of a pre-equilibrated POPC lipid bilayer containing 512 lipids, ensuring that the transmembrane helices of the Sec61α overlap with the hydrophobic region of the lipid membrane. Heavy atoms were position-restrained with a harmonic potential and a strong force constant of 10^5^ kJ mol^-1^ nm^-2^ and the inflateGRO tool (Kandt et al., 2007) was executed to rescale the lipid atom coordinates by a factor of four. A series of 25 iterations of shrinking and energy minimizing was performed until the POPC area per lipid was 71 Å^2^, a little above the equilibrium area per lipid of 65.8 Å^2^ (Tieleman et al., 1998). The system was subsequently solvated and any water molecules inserted in the lipid phase were deleted. NaCl was added to neutralize the system and to provide a physiological ion concentration of 0.15 M.

Molecular dynamics simulations were performed using the GROMACS software package, version 2019 (Van Der Spoel et al., 2005). Initially, the system was energy minimized with the steepest descent algorithm until the maximum force did not exceed 100 kJ mol^-1^ nm^-1^. A short equilibration run in the NVT ensemble was carried out for 5 ns and the temperature was stabilized at 310 K with the velocity-rescale thermostat (Bussi et al., 2007) and a coupling constant of 0.1 ps. An additional equilibration step was performed subsequently in the NPT ensemble for 10 ns. In this case, the temperature was maintained at 310 K using the Nosé–Hoover thermostat (Hoover, 1985; Nose, 1983a, 1988) and a time constant of 0.5 ps, while the target pressure of 1 bar was reached using the Parrinello-Rahman barostat (Nose and Klein, 1983; Parrinello and Rahman, 1981) and a semi-isotropic coupling with a time constant of 5 ps. For the MD production runs, the protein backbone atoms were position-restrained by applying a soft harmonic potential with a force constant of 500 kJ mol^-1^ nm^-2^, in order to ensure that the Sec61 state obtained from electron microscopy would be maintained despite the absence of the ribosome during simulations. Periodic boundary conditions were applied in all three dimensions. The hydrogen-containing bonds were constrained using the LINCS algorithm (Hess, 2008). Neighbor searching, Lennard-Jones interactions and real-space Coulomb interactions were cut off at 10 Å. A dispersion correction was applied to energy and pressure to account for truncation of Lennard-Jones interactions. The particle-mesh Ewald summation was adopted for treatment of reciprocal-space electrostatic interactions with a cubic interpolation and a grid spacing of 1.2 Å. The time step was 2 fs and the leap-frog algorithm was selected for integration of the equations of motion. Coordinates were saved every 10 ps. Three repeats of 100 ns-long MD runs were subsequently performed for each system, amounting to a total simulation time of 600 ns.

To investigate the effect of mutations on mycolactone stability, three mutant systems were generated (S71F, G80W and S82F) using the mutagenesis tool of PyMOL (The PyMOL Molecular Graphics System, Version 1.8 Schrödinger, LLC.). The starting configuration was again the model derived from electron cryo-microscopy, with mycolactone docked in the original conformation. For system setup, equilibration and production runs, the same parameters and procedure as described above were adopted, with the exception that for production runs, position restraints were applied only to the heavy atoms of the Sec61γ subunit and the transmembrane helices H6-H10 of Sec61α to allow the rest of Sec61α to adapt to conformational changes caused by the presence of the mutation. For each mutant system and the WT, three 100 ns-long production runs were performed, resulting in a total simulation time of 1.2 μs.

The VMD software (Humphrey et al., 1996) was used for visualization and the MDAnalysis package (Michaud-Agrawal et al., 2011) for trajectory analysis.

#### Forward genetic screen and viability assays

The forward genetic screen in DNA damage repair-resistant HCT-116 cells was performed as described (Junne et al., 2015; Ogbechi et al., 2018). The mutagen was ethyl methane sulphonate and selection was with 10 ng/ml (13.5 nM) mycolactone A/B. The cDNA for SEC61A1 was sequenced in resistant clones by RT-PCR. Total RNA (1 μg) was reverse transcribed using Superscript IV reverse transcriptase (Life Technologies) according to the manufacturer’s instructions. The coding regions of SEC61A1 were amplified in four overlapping fragments using primers Sec61A1_frag1F/ Sec61A1_frag1R, Sec61A1_frag2F/ Sec61A1_frag2R, Sec61A1_frag3F/ Sec61A1_frag3R, Sec61A1_frag4F/ Sec61A1_frag4R with GoTaq G2 Green Mastermix (Promega). In some cases, confirmation of mutations used an additional fifth fragment using primers Sec61A1_frag5F/ Sec61A1_frag5R. The cycling conditions were 95ᵒC for 2 min, 35x cycles of 95ᵒC for 30 s, 58ᵒC for 30 s and 72ᵒC for 1 min, then 5 min at 72ᵒC. After clean-up, 70ng of each PCR product along with the corresponding forward primer were sent for Sanger sequencing using the Eurofins Mix2Seq service. Returned sequences, covering the entire coding region were analyzed using Chromas and BLASTn to identify the mutations. No homozygous mutations were identified. The parental cells and all resistant cell lines were heterozygous for a silent C/T transition in Thr445 (ACC/ACT). Almost all clones analyzed showed heterozygous mutations of single alleles. One exception was Hct7 which showed both Q127K and S82Y. Hct30 carried R66K and potentially P140T (as yet unconfirmed).

For viability assays, cells were seeded at 1000 cells/well and then treated with mycolactone, cotransin (Mackinnon et al., 2014) (CT8: kind gift of Jack Taunton), or ipomoeassin F (Cao et al., 2007) (kind gift of Wei Shi) for 5 days. Then resazurin was added to a final concentration of 0.01 mg/ml. After 4 h further incubation, viability was measured as emission at 620 nm at excitation 580 nm using a BMG Fluostar Optima Microplate Reader. Data are expressed as a viability index, representing the relative signal compared to the negative control (DMSO-treated cells).

#### Generation of mycolactone resistant cell lines

Synthetic C-terminal Flag-tagged Sec61A1 genes containing the mutations S71F, G80W and S85Y were generated (Thermo Fisher GeneArt) and cloned into the pNLF1-C vector (Promega), replacing the nanoluciferase insert. The plasmid was digested with *MluI* and 2 μg transfected into TRex-293 cells (Thermo Fisher) with Fugene (Promega) according to the manufacturer’s instructions. Cells were selected for one week in the presence of 10 ng/ml (13.5 nM) mycolactone epimers (kind gift from Dr Nicolas Blanchard, CRNS France) (Saint-Auret et al., 2017a, Saint-Auret et al., 2017b) and then for 1 week with 600 μg/ml hygromycin (Thermo Fisher). Expression of the mutant gene was confirmed by immunoblotting, and/or immunofluorescence, for the Flag tag. Single clones were selected for analysis.

#### Immunofluorescence

Cells were transiently transfected with plasmids containing mutant Sec61 genes, incubated overnight, then seeded onto coverslips. After a further 24 hr incubation, cells were fixed with 4% paraformaldehyde in PBS and blocked with 1% BSA/PBS. Coverslips were incubated with anti-Flag antibody followed by Alex488 conjugated anti-rabbit antibody, then counterstained with DAPI.

#### Microsome preparation

Crude rough microsomes were prepared from cells by the method described in Chitwood et al. (2018) with minor variations. Briefly, wild-type TRex-293 cells and those clones overexpressing Sec61α mutants were grown to 70%–90% confluency on eight 500 cm^2^ plates, collected in ice-cold PBS using cell lifters (Costar), centrifuged at 500 x g for 5 min at 4°C, and washed twice in ice-cold PBS. The cell pellet was resuspended in 3 pellet volumes of ice-cold buffer (10 mM HEPES, pH 7.4, 250 mM sucrose, 2 mM MgOAc). Cells were lysed in the cold (4°C) by 25 passes through a 25-gauge needle using a 10 mL syringe. The lysates were centrifuged at 3,800 x g for 30 min at 4°C. The supernatant was centrifuged at 75,000 x g for 1 h at 4°C in an SW55Ti rotor (Beckman Coulter). The membrane pellet was resuspended in microsome buffer (10 mM HEPES, pH 7.4, 250 mM sucrose, 1 mM DTT). Aliquots were taken at each stage for analysis by immunoblotting and assessment of absorbance at 260 nm. RTCs were isolated from cell derived microsomal preparations as described above for CPMM.

#### Figure preparation

Figures were prepared using the programs Chimera (Goddard et al., 2007), ChimeraX (Goddard et al., 2018) and GraphPad Prism versions 7.0 or 8.2.1 (GraphPad Software).

### Quantification and Statistical Analysis

For mycolactone quantification by LC-MS, the calibration curve was established using the mean of the three repeat runs and the slope and intercept were calculated (Microsoft Excel 2016) for interpolation of mycolactone concentrations in samples. In order to compare TRex-293 cells to those overexpressing Sec61α mutants, the absorbance at 260 nm was used to estimate the concentration of RTCs in the recovered samples, using a μM extinction coefficient of 60.8 (Collins and Gilmore, 1991), for the 80S ribosome. The ratio of mycolactone:RTCs was normalized to wild-type cells. Alternatively, the pixel intensity of the Sec61α band from immunoblots of equal volumes of recovered RTCs (measured using ImageJ v.1.52p) was used to estimate the normalized relative ratio. Here, the individual data from the triplicate measurements from a single run is shown. Since the endogenous Sec61α was not depleted from the cells prior to the analysis, all preparations contain some wild-type Sec61α.

For quantification of cell viability, each biological repeat was performed in triplicate. The data were normalized to the mean of the absorbance at 620 nm for DMSO-treated cells (solvent control). When combining the data from independent biological repeats, the mean of each normalized technical triplicate was taken. The IC_50_ for wild-type cells was calculated using log(inhibitor) versus response – variable slope (four parameter) using Graphpad v. 8.2.1. No slopes were fitted to the resistant clones, as the fit was ambiguous, and no statistical comparison was performed. In the figure legends, n is the number of biological repeats (independent experiments).

## Supplemental Information

Document S1. Figures S1–S9 and Tables S1 and S2

Document S2. Article plus Supplemental Information

## References

[bib1] Adams P.D., Afonine P.V., Bunkóczi G., Chen V.B., Davis I.W., Echols N., Headd J.J., Hung L.W., Kapral G.J., Grosse-Kunstleve R.W. (2010). PHENIX: a comprehensive Python-based system for macromolecular structure solution. Acta Crystallogr. D Biol. Crystallogr..

[bib2] Baron L., Paatero A.O., Morel J.D., Impens F., Guenin-Macé L., Saint-Auret S., Blanchard N., Dillmann R., Niang F., Pellegrini S. (2016). Mycolactone subverts immunity by selectively blocking the Sec61 translocon. J. Exp. Med..

[bib3] Bayley C.I., Cieplak P., Cornell W., Kollman P.A. (1993). A well-behaved electrostatic potential based method using charge restraints for deriving atomic charges: the RESP model. J. Phys. Chem..

[bib4] Besemer J., Harant H., Wang S., Oberhauser B., Marquardt K., Foster C.A., Schreiner E.P., de Vries J.E., Dascher-Nadel C., Lindley I.J. (2005). Selective inhibition of cotranslational translocation of vascular cell adhesion molecule 1. Nature.

[bib5] Bussi G., Donadio D., Parrinello M. (2007). Canonical sampling through velocity rescaling. J. Chem. Phys..

[bib6] Cao S., Norris A., Wisse J.H., Miller J.S., Evans R., Kingston D.G. (2007). Ipomoeassin F, a new cytotoxic macrocyclic glycoresin from the leaves of Ipomoea squamosa from the Suriname rainforest. Nat. Prod. Res..

[bib7] Chitwood P.J., Juszkiewicz S., Guna A., Shao S., Hegde R.S. (2018). EMC Is Required to Initiate Accurate Membrane Protein Topogenesis. Cell.

[bib8] Collins P.G., Gilmore R. (1991). Ribosome binding to the endoplasmic reticulum: a 180-kD protein identified by crosslinking to membrane-bound ribosomes is not required for ribosome binding activity. J. Cell Biol..

[bib9] Cross B.C., McKibbin C., Callan A.C., Roboti P., Piacenti M., Rabu C., Wilson C.M., Whitehead R., Flitsch S.L., Pool M.R. (2009). Eeyarestatin I inhibits Sec61-mediated protein translocation at the endoplasmic reticulum. J. Cell Sci..

[bib10] Demangel C., High S. (2018). Sec61 blockade by mycolactone: A central mechanism in Buruli ulcer disease. Biol. Cell.

[bib11] Elmlund D., Elmlund H. (2012). SIMPLE: Software for ab initio reconstruction of heterogeneous single-particles. J. Struct. Biol..

[bib12] Emsley P., Cowtan K. (2004). Coot: model-building tools for molecular graphics. Acta Crystallogr. D Biol. Crystallogr..

[bib13] Garrison J.L., Kunkel E.J., Hegde R.S., Taunton J. (2005). A substrate-specific inhibitor of protein translocation into the endoplasmic reticulum. Nature.

[bib14] Gehringer M., Mäder P., Gersbach P., Pfeiffer B., Scherr N., Dangy J.P., Pluschke G., Altmann K.H. (2019). Configurationally Stabilized Analogs of *M. ulcerans* Exotoxins Mycolactones A and B Reveal the Importance of Side Chain Geometry for Mycolactone Virulence. Org. Lett..

[bib15] Gemmer M., Förster F. (2020). A clearer picture of the ER translocon complex. J. Cell Sci..

[bib16] George K.M., Chatterjee D., Gunawardana G., Welty D., Hayman J., Lee R., Small P.L. (1999). Mycolactone: a polyketide toxin from Mycobacterium ulcerans required for virulence. Science.

[bib17] Goddard T.D., Huang C.C., Ferrin T.E. (2007). Visualizing density maps with UCSF Chimera. J. Struct. Biol..

[bib18] Goddard T.D., Huang C.C., Meng E.C., Pettersen E.F., Couch G.S., Morris J.H., Ferrin T.E. (2018). UCSF ChimeraX: Meeting modern challenges in visualization and analysis. Protein Sci..

[bib19] Gouridis G., Karamanou S., Gelis I., Kalodimos C.G., Economou A. (2009). Signal peptides are allosteric activators of the protein translocase. Nature.

[bib20] Hall B.S., Hill K., McKenna M., Ogbechi J., High S., Willis A.E., Simmonds R.E. (2014). The pathogenic mechanism of the Mycobacterium ulcerans virulence factor, mycolactone, depends on blockade of protein translocation into the ER. PLoS Pathog..

[bib21] Haßdenteufel S., Johnson N., Paton A.W., Paton J.C., High S., Zimmermann R. (2018). Chaperone-Mediated Sec61 Channel Gating during ER Import of Small Precursor Proteins Overcomes Sec61 Inhibitor-Reinforced Energy Barrier. Cell Rep..

[bib22] Hess B. (2008). P-LINCS: A Parallel Linear Constraint Solver for Molecular Simulation. J. Chem. Theory Comput..

[bib23] Hong H., Gates P.J., Staunton J., Stinear T., Cole S.T., Leadlay P.F., Spencer J.B. (2003). Identification using LC-MSn of co-metabolites in the biosynthesis of the polyketide toxin mycolactone by a clinical isolate of Mycobacterium ulcerans. Chem. Commun. (Camb.).

[bib24] Hoover W.G. (1985). Canonical dynamics: Equilibrium phase-space distributions. Phys. Rev. A Gen. Physiol..

[bib25] Humphrey W., Dalke A., Schulten K. (1996). VMD: visual molecular dynamics. J. Mol. Graph..

[bib26] Itskanov S., Park E. (2019). Structure of the posttranslational Sec protein-translocation channel complex from yeast. Science.

[bib27] Jämbeck J.P., Lyubartsev A.P. (2012). Derivation and systematic validation of a refined all-atom force field for phosphatidylcholine lipids. J. Phys. Chem. B.

[bib28] Jämbeck J.P., Lyubartsev A.P. (2012). An Extension and Further Validation of an All-Atomistic Force Field for Biological Membranes. J. Chem. Theory Comput..

[bib29] Jämbeck J.P., Lyubartsev A.P. (2013). Another Piece of the Membrane Puzzle: Extending Slipids Further. J. Chem. Theory Comput..

[bib30] Jorgensen W.L., Chandrasekhar J., Madura J.D. (1983). Comparison of simple potential functions for simulating liquid water. J. Chem. Phys..

[bib31] Junne T., Schwede T., Goder V., Spiess M. (2007). Mutations in the Sec61p channel affecting signal sequence recognition and membrane protein topology. J. Biol. Chem..

[bib32] Junne T., Wong J., Studer C., Aust T., Bauer B.W., Beibel M., Bhullar B., Bruccoleri R., Eichenberger J., Estoppey D. (2015). Decatransin, a new natural product inhibiting protein translocation at the Sec61/SecYEG translocon. J. Cell Sci..

[bib33] Kandt C., Ash W.L., Tieleman D.P. (2007). Setting up and running molecular dynamics simulations of membrane proteins. Methods.

[bib34] Kelkar A., Dobberstein B. (2009). Sec61beta, a subunit of the Sec61 protein translocation channel at the endoplasmic reticulum, is involved in the transport of Gurken to the plasma membrane. BMC Cell Biol..

[bib35] Lang S., Pfeffer S., Lee P.H., Cavalié A., Helms V., Förster F., Zimmermann R. (2017). An Update on Sec61 Channel Functions, Mechanisms, and Related Diseases. Front. Physiol..

[bib36] Lindorff-Larsen K., Piana S., Palmo K., Maragakis P., Klepeis J.L., Dror R.O., Shaw D.E. (2010). Improved side-chain torsion potentials for the Amber ff99SB protein force field. Proteins.

[bib37] Liu K., Watanabe E., Kokubo H. (2017). Exploring the stability of ligand binding modes to proteins by molecular dynamics simulations. J. Comput. Aided Mol. Des..

[bib38] López C.A., Unkefer C.J., Swanson B.I., Swanson J.M.J., Gnanakaran S. (2018). Membrane perturbing properties of toxin mycolactone from Mycobacterium ulcerans. PLoS Comput. Biol..

[bib39] MacKinnon A.L., Garrison J.L., Hegde R.S., Taunton J. (2007). Photo-leucine incorporation reveals the target of a cyclodepsipeptide inhibitor of cotranslational translocation. J. Am. Chem. Soc..

[bib40] Mackinnon A.L., Paavilainen V.O., Sharma A., Hegde R.S., Taunton J. (2014). An allosteric Sec61 inhibitor traps nascent transmembrane helices at the lateral gate. eLife.

[bib41] McKenna M., Simmonds R.E., High S. (2016). Mechanistic insights into the inhibition of Sec61-dependent co- and post-translational translocation by mycolactone. J. Cell Sci..

[bib42] McKenna M., Simmonds R.E., High S. (2017). Mycolactone reveals the substrate-driven complexity of Sec61-dependent transmembrane protein biogenesis. J. Cell Sci..

[bib43] Michaud-Agrawal N., Denning E.J., Woolf T.B., Beckstein O. (2011). MDAnalysis: a toolkit for the analysis of molecular dynamics simulations. J. Comput. Chem..

[bib44] Nose S. (1983). A molecular dynamics method for simulations in the canonical ensemble. Mol. Phys..

[bib45] Nose S. (1988). A unified formulation of the constant temperature molecular dynamics methods. J. Chem. Phys..

[bib46] Nose S., Klein M.L. (1983). A study of solid and liquid carbon tetrafluoride using the constant pressure molecular dynamics technique. J. Chem. Phys..

[bib47] Ogbechi J., Hall B.S., Sbarrato T., Taunton J., Willis A.E., Wek R.C., Simmonds R.E. (2018). Inhibition of Sec61-dependent translocation by mycolactone uncouples the integrated stress response from ER stress, driving cytotoxicity via translational activation of ATF4. Cell Death Dis..

[bib48] Paatero A.O., Kellosalo J., Dunyak B.M., Almaliti J., Gestwicki J.E., Gerwick W.H., Taunton J., Paavilainen V.O. (2016). Apratoxin Kills Cells by Direct Blockade of the Sec61 Protein Translocation Channel. Cell Chem. Biol..

[bib49] Parrinello M., Rahman A. (1981). Polymorphic transitions in single crystals: a new molecular dynamics method. J. Appl. Phys..

[bib50] Pettersen E.F., Goddard T.D., Huang C.C., Couch G.S., Greenblatt D.M., Meng E.C., Ferrin T.E. (2004). UCSF Chimera--a visualization system for exploratory research and analysis. J. Comput. Chem..

[bib51] Rapoport T.A., Li L., Park E. (2017). Structural and Mechanistic Insights into Protein Translocation. Annu. Rev. Cell Dev. Biol..

[bib52] Rohou A., Grigorieff N. (2015). CTFFIND4: Fast and accurate defocus estimation from electron micrographs. J. Struct. Biol..

[bib53] Saint-Auret S., Abdelkafi H., Le Nouen D., Bisseret P., Blanchard N. (2017). Synthetic strategies towards mycolactone A/B, an exotoxin secreted by Mycobacterium ulcerans. Org. Chem. Front..

[bib54] Saint-Auret S., Abdelkafi H., Le Nouen D., Guenin-Macé L., Demangel C., Bisseret P., Blanchard N. (2017). Modular total syntheses of mycolactone A/B and its [^2^H]-isotopologue. Org. Biomol. Chem..

[bib55] Scheres S.H. (2012). RELION: implementation of a Bayesian approach to cryo-EM structure determination. J. Struct. Biol..

[bib56] Scheres S.H., Chen S. (2012). Prevention of overfitting in cryo-EM structure determination. Nat. Methods.

[bib57] Schubert D., Klein M.C., Hassdenteufel S., Caballero-Oteyza A., Yang L., Proietti M., Bulashevska A., Kemming J., Kühn J., Winzer S. (2018). Plasma cell deficiency in human subjects with heterozygous mutations in Sec61 translocon alpha 1 subunit (SEC61A1). J. Allergy Clin. Immunol..

[bib58] Smith M.A., Clemons W.M., DeMars C.J., Flower A.M. (2005). Modeling the effects of prl mutations on the Escherichia coli SecY complex. J. Bacteriol..

[bib59] Song F., Fidanze S., Benowitz A.B., Kishi Y. (2002). Total synthesis of the mycolactones. Org. Lett..

[bib60] Swint-Kruse L., Brown C.S. (2005). Resmap: automated representation of macromolecular interfaces as two-dimensional networks. Bioinformatics.

[bib61] Tieleman D.P., Forrest L.R., Sansom M.S., Berendsen H.J. (1998). Lipid properties and the orientation of aromatic residues in OmpF, influenza M2, and alamethicin systems: molecular dynamics simulations. Biochemistry.

[bib62] Trueman S.F., Mandon E.C., Gilmore R. (2012). A gating motif in the translocation channel sets the hydrophobicity threshold for signal sequence function. J. Cell Biol..

[bib63] Van den Berg B., Clemons W.M., Collinson I., Modis Y., Hartmann E., Harrison S.C., Rapoport T.A. (2004). X-ray structure of a protein-conducting channel. Nature.

[bib64] Van Der Spoel D., Lindahl E., Hess B., Groenhof G., Mark A.E., Berendsen H.J. (2005). GROMACS: fast, flexible, and free. J. Comput. Chem..

[bib65] Van Puyenbroeck V., Vermeire K. (2018). Inhibitors of protein translocation across membranes of the secretory pathway: novel antimicrobial and anticancer agents. Cell. Mol. Life Sci..

[bib66] Voorhees R.M., Hegde R.S. (2016). Structure of the Sec61 channel opened by a signal sequence. Science.

[bib67] Voorhees R.M., Hegde R.S. (2016). Toward a structural understanding of co-translational protein translocation. Curr. Opin. Cell Biol..

[bib68] Voorhees R.M., Fernández I.S., Scheres S.H., Hegde R.S. (2014). Structure of the mammalian ribosome-Sec61 complex to 3.4 Å resolution. Cell.

[bib69] Walter P., Blobel G. (1983). Preparation of microsomal membranes for cotranslational protein translocation. Methods Enzymol..

[bib70] Wang J., Wolf R.M., Caldwell J.W., Kollman P.A., Case D.A. (2004). Development and testing of a general amber force field. J. Comput. Chem..

[bib71] Wu X., Cabanos C., Rapoport T.A. (2019). Structure of the post-translational protein translocation machinery of the ER membrane. Nature.

[bib72] Yotsu R.R., Suzuki K., Simmonds R.E., Bedimo R., Ablordey A., Yeboah-Manu D., Phillips R., Asiedu K. (2018). Buruli Ulcer: a Review of the Current Knowledge. Curr. Trop. Med. Rep..

[bib73] Zheng S.Q., Palovcak E., Armache J.P., Verba K.A., Cheng Y., Agard D.A. (2017). MotionCor2: anisotropic correction of beam-induced motion for improved cryo-electron microscopy. Nat. Methods.

[bib74] Zivanov J., Nakane T., Forsberg B.O., Kimanius D., Hagen W.J., Lindahl E., Scheres S.H. (2018). New tools for automated high-resolution cryo-EM structure determination in RELION-3. eLife.

[bib75] Zong G., Hu Z., O’Keefe S., Tranter D., Iannotti M.J., Baron L., Hall B., Corfield K., Paatero A.O., Henderson M.J. (2019). Ipomoeassin F Binds Sec61α to Inhibit Protein Translocation. J. Am. Chem. Soc..

