# Supplementary material for: Structure of the Inhibited State of the Sec Translocon
Source: Mol Cell. 2020 Aug 6;79(3):406–415.e7. doi: 10.1016/j.molcel.2020.06.013 (PMC7427319; doi:10.1016/j.molcel.2020.06.013)
Supplement: Document S1. Figures S1–S9 and Tables S1 and S2 [file mmc1.pdf]

Molecular Cell, Volume *79*

Supplemental Information

Structure of the Inhibited State of the Sec Translocon

Samuel F. Gérard, Belinda S. Hall, Afroditi M. Zaki, Katherine A. Corfield, Peter U. Mayerhofer, Catia Costa, Daniel K. Whelligan, Philip C. Biggin, Rachel E. Simmonds, and Matthew K. Higgins

Supplementary Information

Structure of the inhibited state of the Sec translocon

Samuel F. Gérard, Belinda S. Hall, Afroditi Zaki, Katherine A. Corfield, Peter U. Mayerhofer, Catia Costa, Daniel K. Whelligan, Philip C Biggin, Rachel E. Simmonds and Matthew K. Higgins

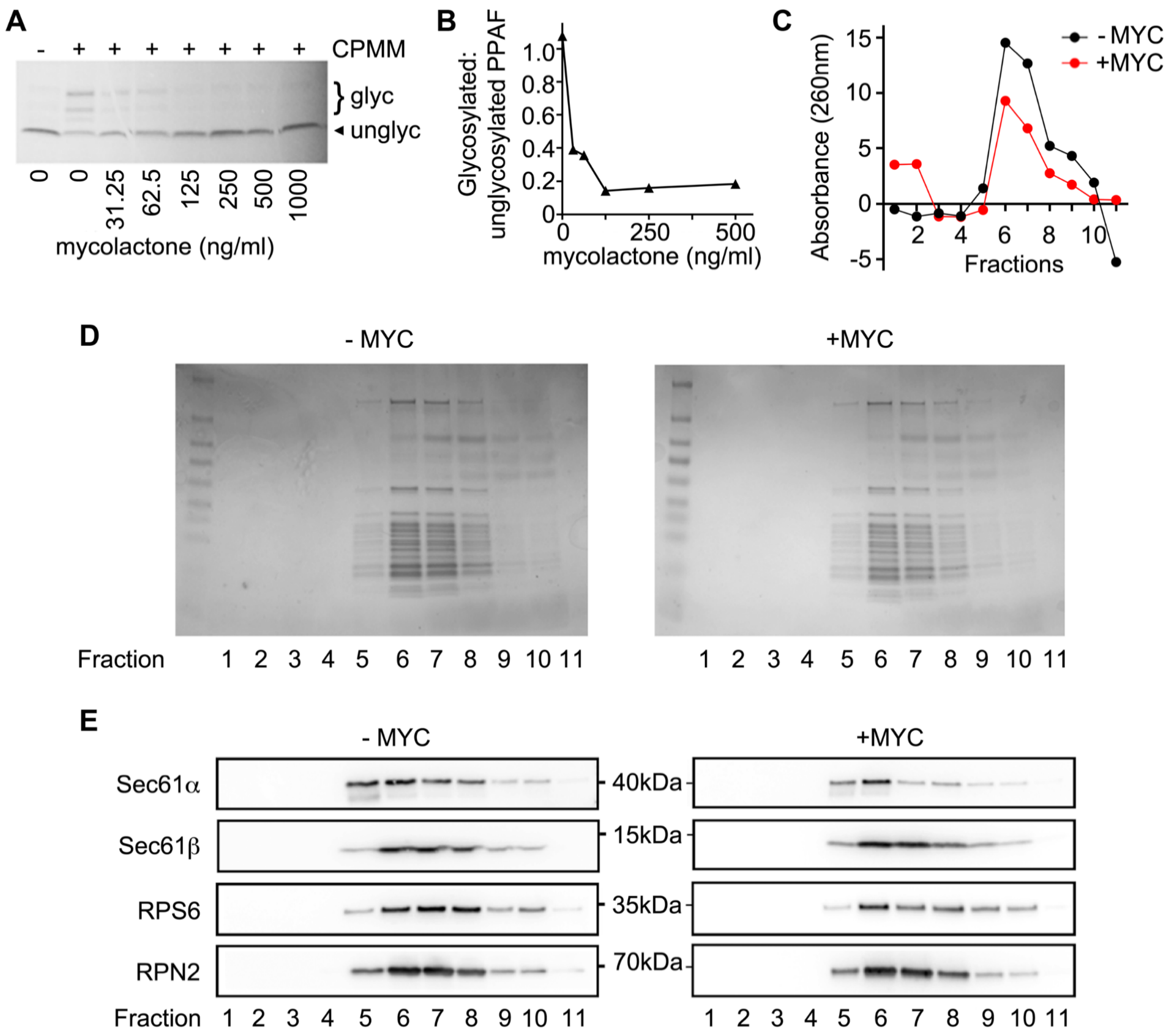

Figure S1. Preparation of ribosome-translocon complexes (related to Figure 1)

A and **B**. The concentration of mycolactone that fully inhibits translocation of yeast prepro-α factor (PPAF) by the canine pancreatic microsomal membranes (CPMM) used in this study was determined empirically by an *in vitro* translation/translocation assay and is expressed as the ratio of total glycosylated (glyc) to un-glycosylated (unglyc) protein. Translocation was maximally inhibited at 125 ng/ml (168.2 nM).

A. Phosphorimage of 35-S methionine labelled proteins separated by SDS-PAGE.

B. Quantitation of the ratio of glycosylated and unglycosylated PPAF at different concentrations of mycolactone.

C–E. CPMM were nuclease treated and exposed to 0.1% DMSO (- MYC) or 200 ng/ml (269.2 nM) mycolactone in a 0.1% (w/v) BSA carrier (+ MYC) for 30 min on ice. These were solubilised in 1.75% digitonin, and the soluble fraction separated by size exclusion chromatography. A sample from each eluted fraction was separated by SDS-PAGE on 4-20% acrylamide gels.

C. The absorbance of collected fractions at 260 nm

D. Total protein content of fractions monitored by Coomassie staining

E. Immunoblotting of fractions for components of ribosome-translocon complexes; migration relative to known molecular weight markers are shown.

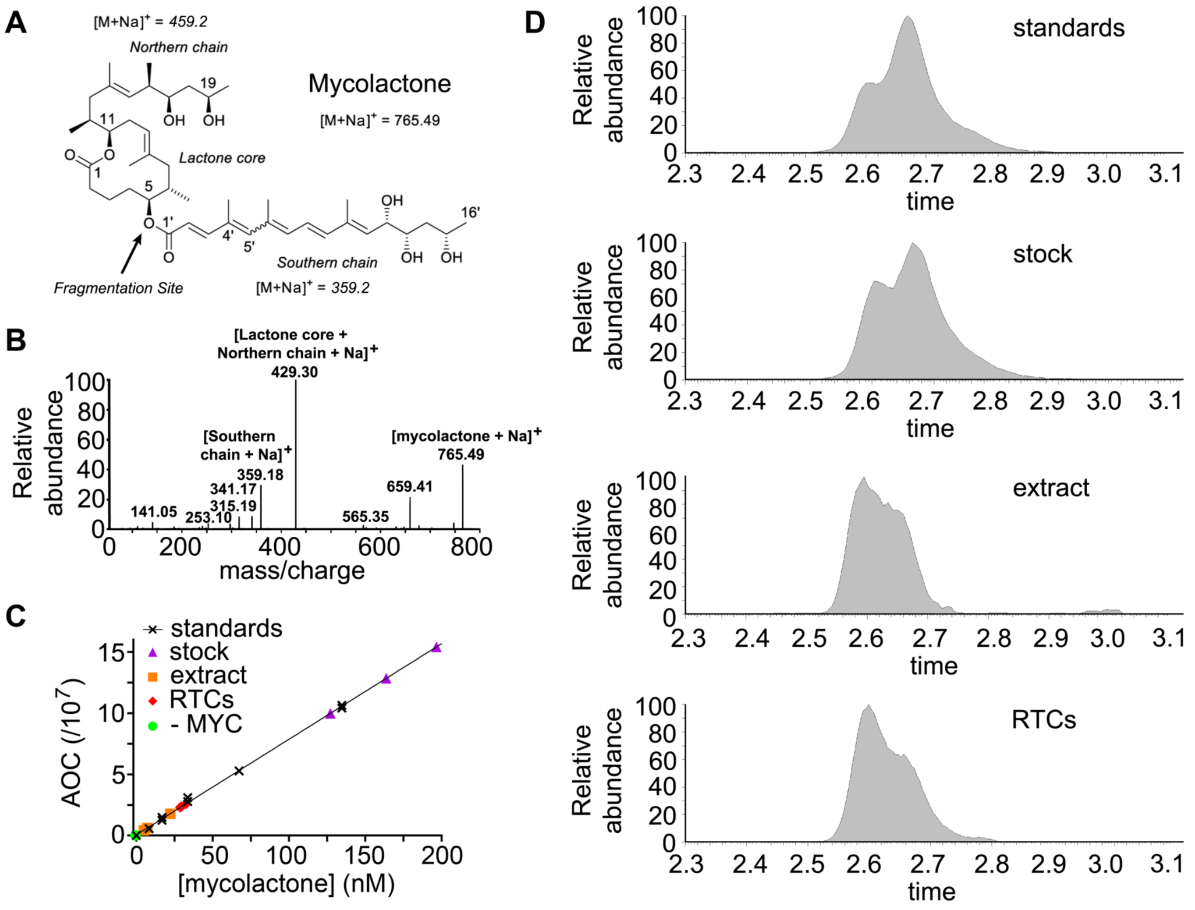

Figure S2. Liquid chromatography-mass spectrometry (LC-MS) of ribosome-translocon complexes and isolation intermediates (related to Figure 1)

A. The structure of mycolactone indicating the expected *m/z* for the $[M+Na]^+$ ions of the whole molecule and MS/MS fragmentation products.

B. The identity and LC-MS retention time of mycolactone was confirmed by both accurate mass and MS/MS of the $[M+Na]^+$ ion, which generated the expected fragmentation pattern of sodium adducts (*m/z* 429.3 and 359.2) (Hong et al., 2003).

C and **D.** Estimation of mycolactone quantities at different stages of sample preparation using a calibration curve of mycolactone and integrating the extracted ion chromatogram (EIC) of the $[M+Na]^+$ (*m/z* 765.4721 – 765.5103) ion eluting at 2.38 – 2.98 min; r^2 = 0.9979. “standards”; synthetic mycolactone in DMSO, “stock”; mycolactone diluted in 0.1% (w/v) BSA prior to addition to CPMM, “extract”; total digitonin extract of CPMM incubated with mycolactone before separation by size exclusion chromatography, “RTCs”; pooled eluted peak fractions containing ribosome-translocon complexes. Parallel samples without mycolactone added (- MYC) were also prepared and analysed.

C. Quantitation of samples. Samples to which mycolactone had not been added (- MYC; samples equivalent to stock, extract and RTCs) all registered values at or below those of blanks that were run between samples and overlap at the origin.

D. EICs for different mycolactone-containing samples. The earlier peak almost certainly corresponds to the *E*-Δ4’,5’-isomer (KH Altmann, personal communication). While both isomers are present in the RTCs, a possible enrichment of the *E*-Δ4’,5’-isomer was observed. Data for samples to which mycolactone had not been added did not register above the baseline.

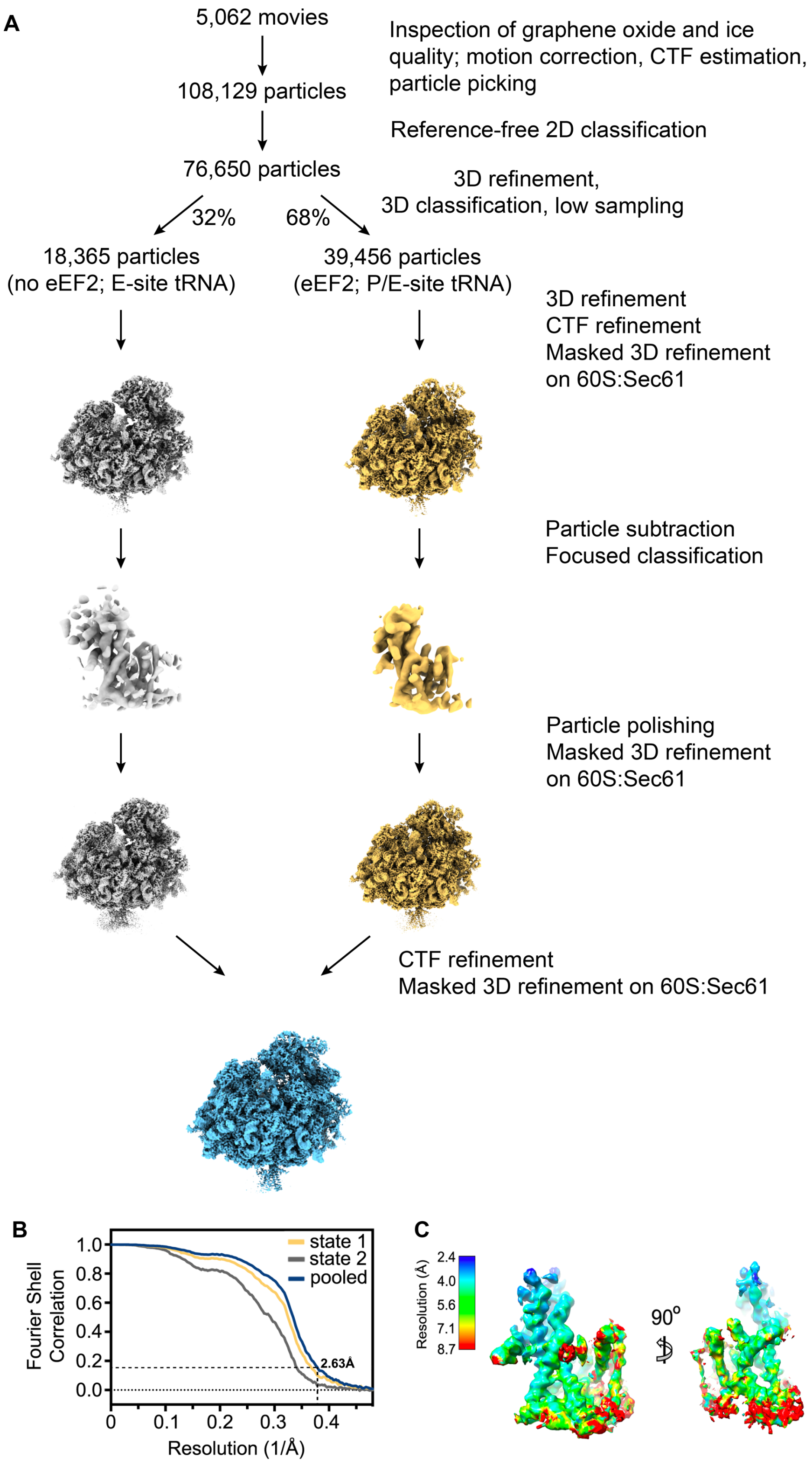

Figure S3: Data collection and image processing information for ribosome-translocon complexes prepared in the presence of mycolactone (related to Figure 1)

A. Data collection and image processing flow chart **B.** Fourier Shell Correlation for state 1 (eEF2 and P/E-site tRNA), state 2 (no eEF2 and E-site tRNA) and pooled particles. A line at FSC=0.143 is shown to indicate a resolution of 2.63 Å for the pooled map. **C.** Representation of the local resolution in the Sec translocon.

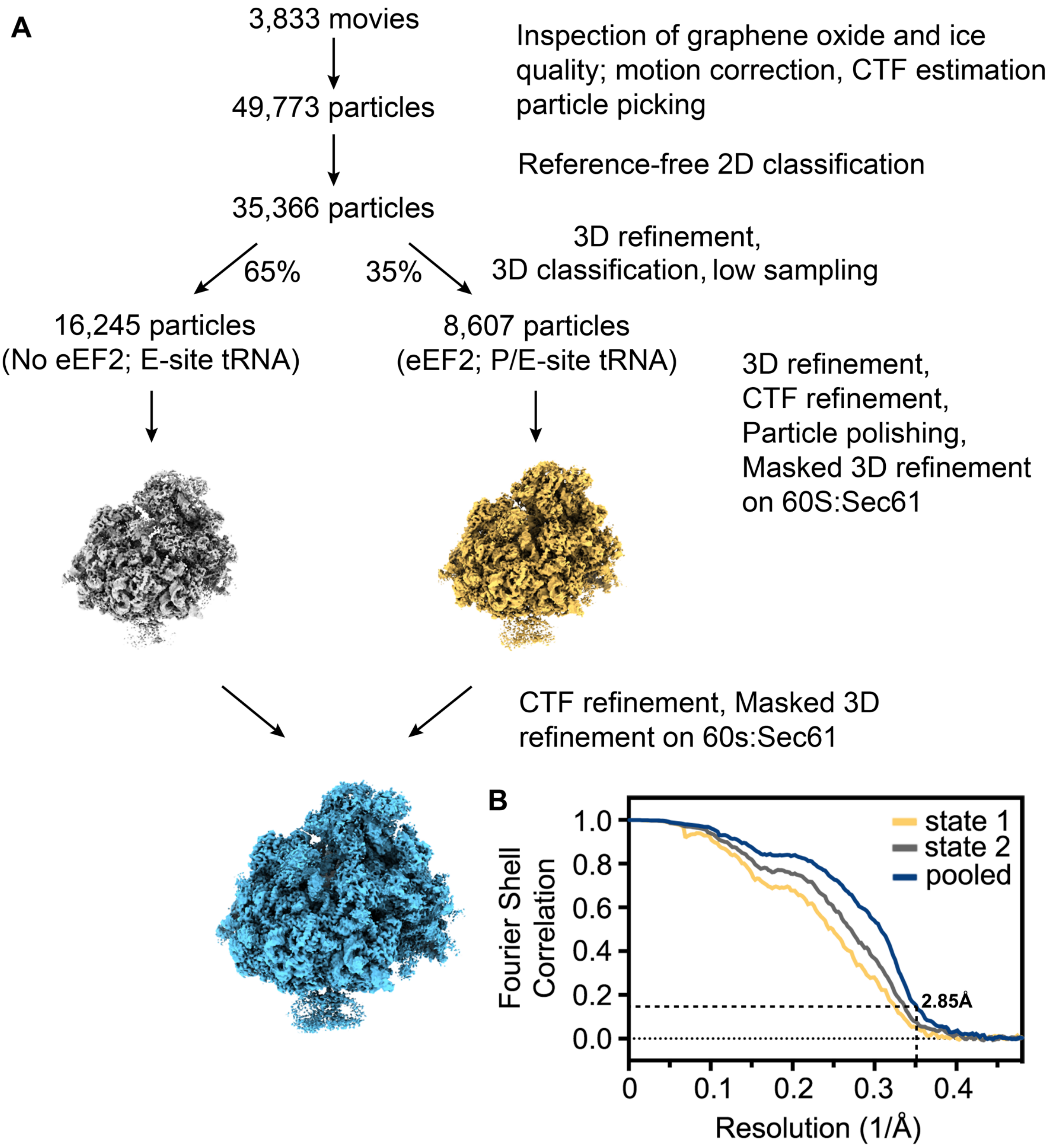

Figure S4: Data collection and image processing flow chart for ribosome-translocon complexes prepared in the absence of mycolactone (related to Figure 1)

A. The data collection and processing scheme.

B. Fourier Shell Correlation for state 1 (eEF2 and P/E-site tRNA), state 2 (no eEF2 and E-site tRNA) and pooled particles. A line at FSC=0.143 is shown to indicate a resolution of 2.85Å for the pooled map.

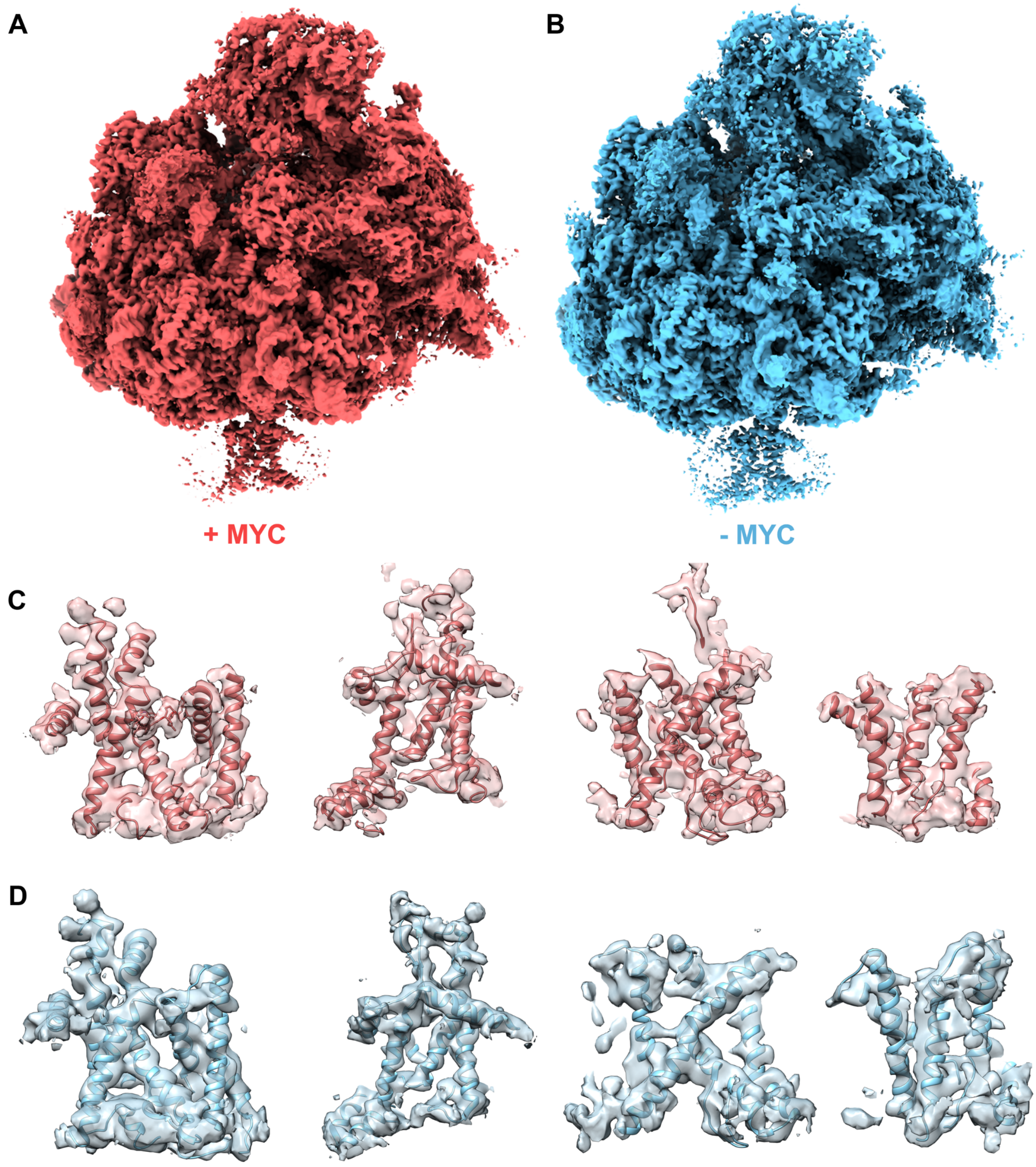

Figure S5: representation of electron densities (related to Figure 1)

A. Electron density for the ribosome-translocon complexes in the presence of mycolactone (+ MYC). **B.** Electron density for the ribosome-translocon complexes in the absence of mycolactone (- MYC). **C.** Electron density for Sec61α in the presence of mycolactone (+MYC). **D.** Electron density for Sec61α in the absence of mycolactone (- MYC). Electron density maps were low-pass filtered to 5Å.

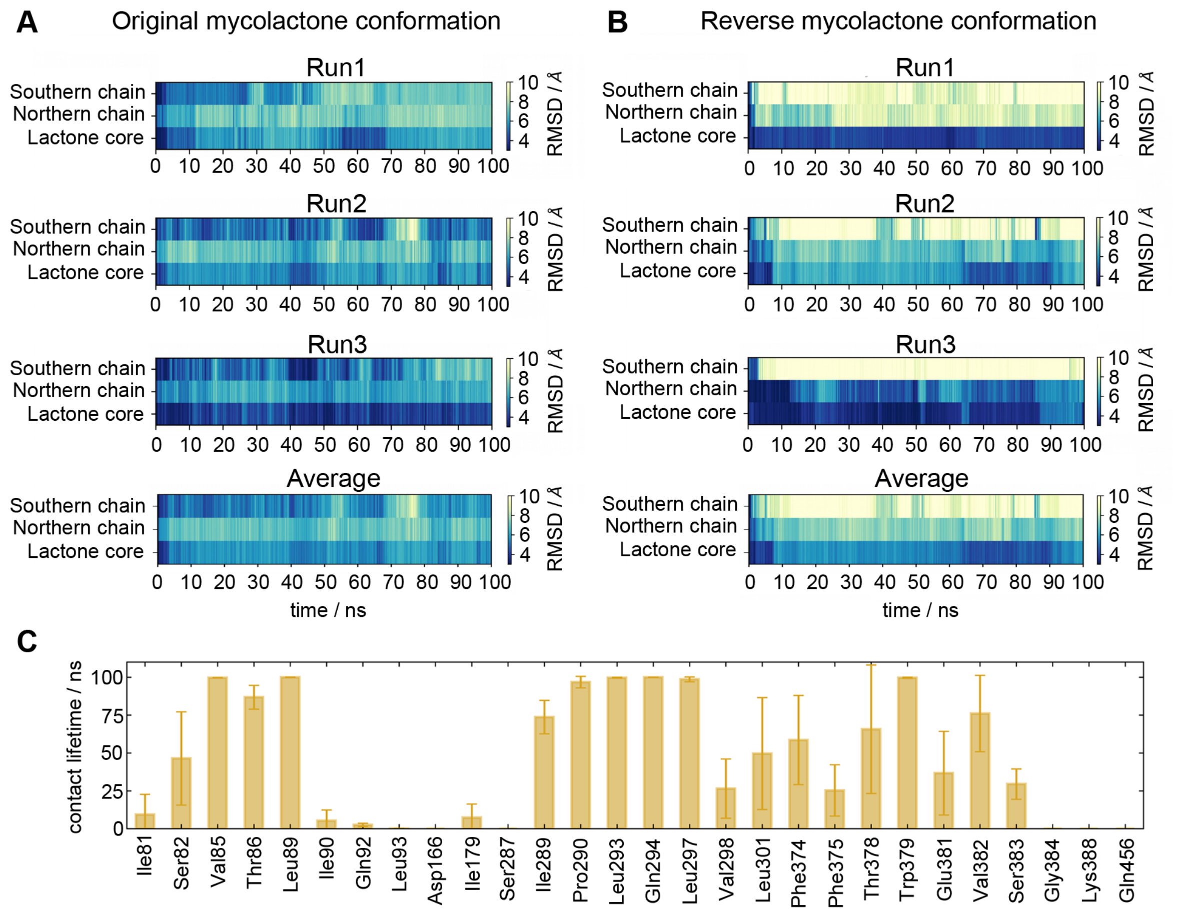

Figure S6: Molecular dynamics simulations to assess mycolactone binding (related to Figure 3)

Assessment of the dynamics of mycolactone during 100 ns simulations run with the *E*-Δ4',5' isomer of mycolactone. **A.** the original conformation, with the southern chain projecting away from the translocon and **B.** the reverse conformation, with the southern chain projecting towards the translocon. In each case, the root mean square deviation from the starting position is shown at each nanosecond for the southern chain, northern chain and lactone core, and the results from three independent simulations are shown, with the final plot showing the average. **C.** The average contact lifetime for each of the residues of Sec61α which contact mycolactone. Contact is defined as being <4 Å from mycolactone. Each bar shows the average of the three independent simulations with error bars corresponding to the standard deviation.

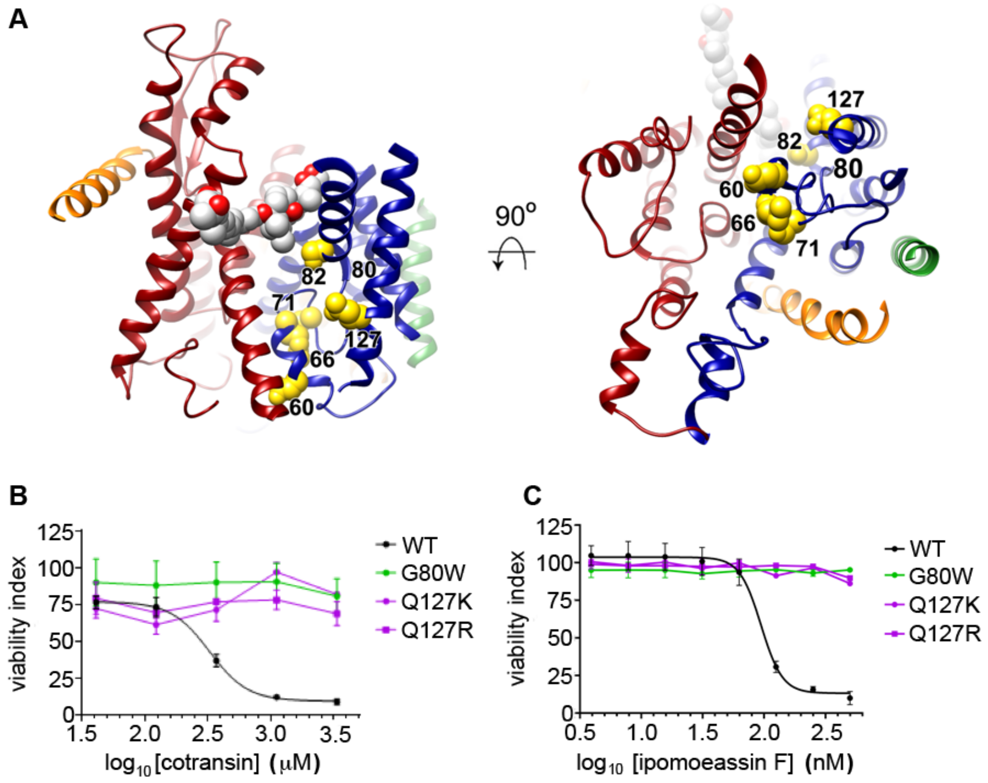

Figure S7: Resistance of mutant clones for translocon inhibitors (related to Figure 4)

A. Two perpendicular views of the Sec translocon with Sec61α coloured with helices H1-H5 in blue and H6-H10 in red, Sec61β in green and Sec61γ in orange. Mycolactone is shown in spheres with carbon in white and oxygen in red. Residues whose mutation leads to mycolactone resistance are represented as yellow spheres.

B. and **C**. Parental HCT-116 cells and representative clones with different amino acid substitutions were tested for their sensitivity to translocation inhibitors **B.** cotransin and **C.** ipomoeassin F. Data is expressed as a normalised viability index of cells treated with inhibitor for 5 days, after which metabolic activity was assessed with Resazurin dye (alamar blue assay) and values normalised to a DMSO control. Data is mean ±SEM of n=3-4 independent experiments

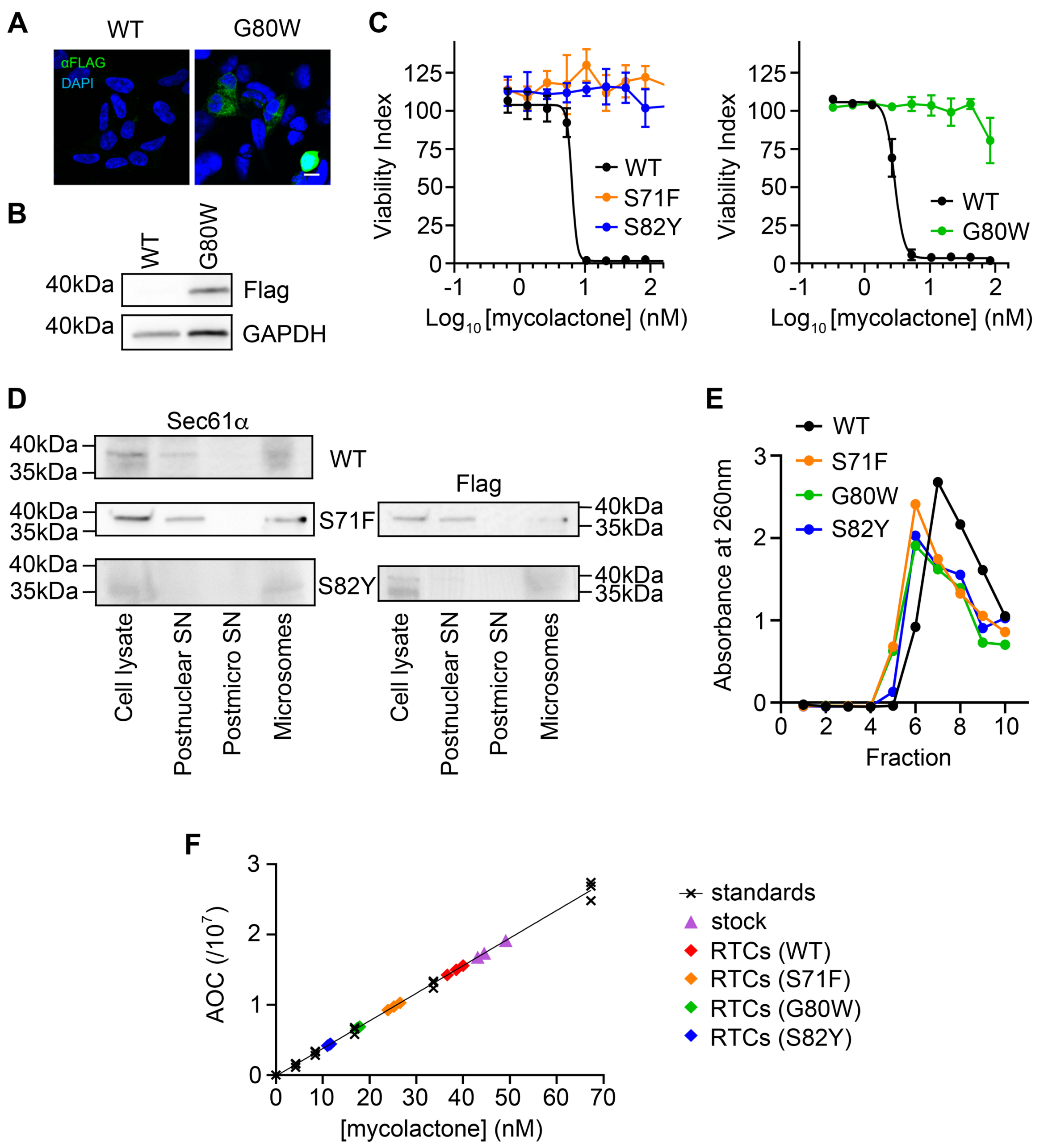

Figure S8: Preparation and analysis of microsomes from cells overexpressing Sec61α resistance mutations (related to Figure 4)

TRex-293 cells were stably transfected with constructs encoding missense mutations (identified in the forward genetic screen) of C-terminal flag-tagged Sec61A1. Several clones were selected with mycolactone A/B epimers for one week and then hygromycin for one week. Data is shown for clones S71F.2, G80W.7 and S82Y.3. Cells were expanded for microsome preparation and investigation of mycolactone binding by LC-MS.

A. Representative data for G80W showing ER membrane incorporation in transiently transfected cells. Immunofluorescence with an anti-flag antibody with a DAPI counterstain.

B. Representative data for G80W showing immunoblotting of stably over-expressing cells. Migration relative to known molecular weight markers are shown.

C. The stable clones were tested for their sensitivity to mycolactone A/B. Data is expressed as a normalised viability index of cells treated with inhibitor for 5 days, after which metabolic

activity was assessed with Resazurin dye (alamar blue assay) and values normalised to a DMSO control. Data is mean ± SD of a triplicate assay performed once (S71F) or twice (S82Y), or mean ± SEM of n=3 (G80W). The IC_{50} for WT cells is 6.3 nM (4.7 ng/ml) and 2.9 nM (2.2 ng/ml) respectively
D. Microsomes were prepared from ~8 m^2 of cells and aliquots from the various stages (including the initial cell lysates and supernatant "SN" of different centrifugation steps), and the resulting microsomes, were analysed by immunoblotting. Migration relative to known molecular weight markers are shown.
E. The cellular microsomes were nuclease treated and exposed to 200 ng/ml (269.2 nM) mycolactone in a 0.1% (w/v) BSA carrier for 30 min on ice. These were solubilised in 1.75% digitonin, and the soluble fraction separated by size exclusion chromatography. The absorbance of collected fractions at 260 nm was assessed.
F. Relative mycolactone abundance in the RTCs was estimated using high resolution LC-MS and integrating the extracted ion chromatogram peaks for $[mycolactone+Na]^+$ (m/z 765.4721 – 765.5103) ion eluting at 2.38 – 2.98 against a calibration curve; r^2 = 0.9963. "standards"; synthetic mycolactone in DMSO diluted into MeCN/H_2O (1:1), "stock"; mycolactone diluted in 0.1% (w/v) BSA prior to addition to microsomes, "RTCs"; eluted peak fractions containing ribosome-translocon complexes.

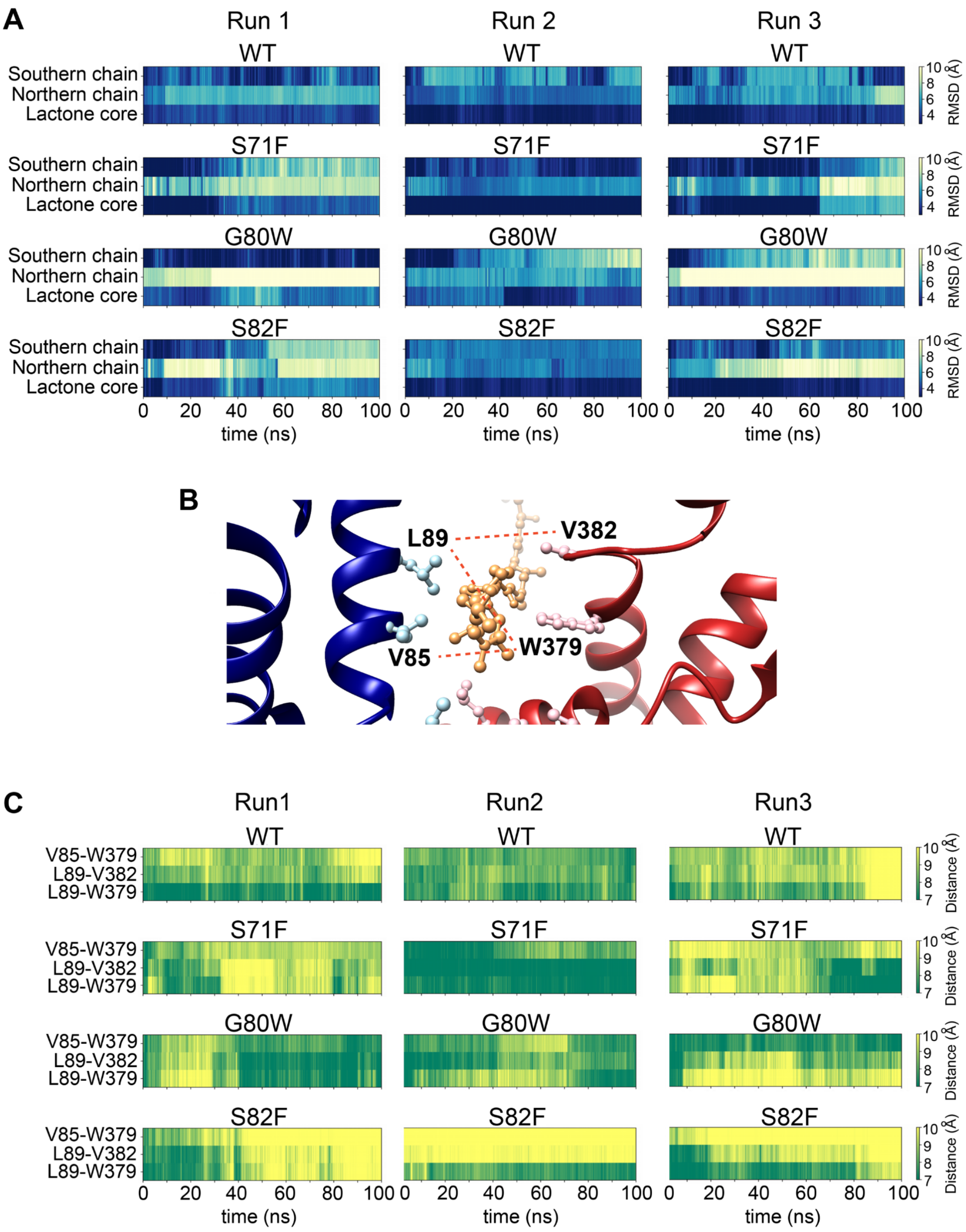

Figure S9: Assessment by molecular dynamics of the effect of resistance mutations on mycolactone binding (related to Figure 4)

A. Assessment of the dynamics of mycolactone bound to wild-type translocon, or to translocons containing resistance mutations, during 100 ns simulations. In each case, helices H6-H10 of the translocon were restrained, while mycolactone and the remainder of the translocon were allowed to move. The root mean square deviation from the starting position is shown at each nanosecond for the southern chain, northern chain and lactone core, and the results from three independent simulations are shown.

B. Structure of the mycolactone binding pocket, showing the positions of V85, L89, V382 and W379.

C. Analysis of the separation of pairs of residues illustrated in **B**, during the simulations described in **A**. In each case, the distance is shown at each nanosecond and the results of three independent simulations are shown.

Table S1. Forward genetic screen of mutations using resistance to mycolactone A/B (related to Figure 4)

Location	WILD TYPE	Properties	Substitutio	MUTANT(S)	Properties	No. of clones	Clone tested
Close to plug helix	D60	Negative charge	GAC -> GGC<br>GAC -> GTC	G60<br>V60	Very small, flexible<br>Non polar, hydrophobic	3<br>1	Hct4[1]
Within plug helix	R66	Positive charge	AGA > GGA<br>AGA -> AAA	G66<br>K66	Very small, flexible<br>Positive charge	1<br>10	Hct6[1]
Close to plug helix	S71	Very small, polar	TCT -> TTT	F71	Bulky, hydrophobic	6	Hct21
H2, lateral gate	G80	Very small, flexible	GGG -> TGG	W80	Bulky, hydrophobic	2	Hct30[2,3]
H2, lateral gate	S82	Very small, polar	TCT -> TTT<br>TCT -> TAT	F82<br>Y82	Bulky, hydrophobic<br>Bulky, hydrophobic	1<br>3	Hct27<br>Hct1
Polar cluster residue	Q127	Polar, charged	CAG -> AAG<br>CAG -> CGG	K127<br>R127	Positive charge<br>Positive charge	3<br>1	Hct2[2]<br>Hct29[2,3]

[1] Previously reported (Ogbechi et al., 2018)
[2] Also tested against cotransin (CT08)
[3] Also tested against ipomoeassin F

Table S2. Conservation of resistance mutations to different classes of translocation inhibitor (related to Figure 4)

Residue	Mutations cause resistance?					*prl*?
	Mycolactone[1]	Cotransin[2]	Decatransin[3]	Apratoxin A[4]	Ipomoeassin F[5]	
Asp60	R	R	R	-	NR	Yes[6]
Arg66	R	R	R	R	R	Yes[6]
Ser71	R	R	R	-	-	Yes[6,7]
Gly80	R	R	-	-	R	Yes[6]
Ser82	R	R	-	R	R	
Gln127	R	R	-	-	R	Yes[6]

[1] Mycolactone A/B; this work and (Baron et al, 2016; Zong et al, 2019 and Ogbechi et al, 2018)
[2] Cotransin or cotransin-like compounds; this work and (MacKinnon et al, 2014; Paatero et al, 2016 and Junne et al, 2015)
[3] See (Junne et al, 2015).
[4] See (Paatero et al, 2016)
[5] Ipomoeassin F; this work and (Zong et al, 2019)
[6] Mutation(s) at structurally equivalent site associated with *prl* phenotype in yeast (Junne et al, 2007; Junne et al, 2015 and Trueman et al, 2012)
[7] Mutation(s) at structurally equivalent site associated with *prl* phenotype in *E. coli* (Smith et al, 2005)
R; resistant (>4-fold increase in IC_{50})
NR; not resistant (<2-fold increase in IC_{50})
-; not determined.
